# Diving deep: zebrafish models in motor neuron degeneration research

**DOI:** 10.3389/fnins.2024.1424025

**Published:** 2024-06-20

**Authors:** Vranda Garg, Bart R. H. Geurten

**Affiliations:** ^1^Department of Cellular Neurobiology, Georg-August-University Göttingen, Göttingen, Lower Saxony, Germany; ^2^Centre de Recherche du Centre Hospitalier de l'Université de Montréal, Montreal, QC, Canada; ^3^Department of Neuroscience, Université de Montréal, Montreal, QC, Canada; ^4^Department of Zoology, University of Otago, Dunedin, New Zealand

**Keywords:** motor neuron disorders, zebrafish, hereditary spastic paraplegia (HSP), amyotrophic lateral sclerosis (ALS), spinal muscular atrophy (SMA), drug screening

## Abstract

In the dynamic landscape of biomedical science, the pursuit of effective treatments for motor neuron disorders like hereditary spastic paraplegia (HSP), amyotrophic lateral sclerosis (ALS), and spinal muscular atrophy (SMA) remains a key priority. Central to this endeavor is the development of robust animal models, with the zebrafish emerging as a prime candidate. Exhibiting embryonic transparency, a swift life cycle, and significant genetic and neuroanatomical congruencies with humans, zebrafish offer substantial potential for research. Despite the difference in locomotion—zebrafish undulate while humans use limbs, the zebrafish presents relevant phenotypic parallels to human motor control disorders, providing valuable insights into neurodegenerative diseases. This review explores the zebrafish's inherent traits and how they facilitate profound insights into the complex behavioral and cellular phenotypes associated with these disorders. Furthermore, we examine recent advancements in high-throughput drug screening using the zebrafish model, a promising avenue for identifying therapeutically potent compounds.

## 1 Introduction

Motor neuron diseases (MNDs) are a clinically diverse group of neurological disorders which are characterized by the progressive degeneration of the upper and lower motor neurons in the brain and spinal cord, respectively. Prominent features of MNDs include muscle spasticity and atrophy, weakness and paralysis of upper and lower body parts affecting speech, swallowing, breathing and movement (Desai and Olney, 2009; Dion et al., 2009; Tiryaki and Horak, 2014; Foster and Salajegheh, 2019). The most common types of MNDs include, hereditary spastic paraplegia (HSP), amyotrophic lateral sclerosis (ALS), and spinal muscular atrophy (SMA). Although these disorders have different etiologies, all of them lead to extreme disability (Khaniani et al., 2013; Robberecht and Philips, 2013; Fink, 2014). Despite considerable progress in comprehending these debilitating disorders, an effective cure remains elusive. This challenge is partly attributed to our incomplete understanding of the pathogenic mechanisms, which are further complicated by mutations in genes regulating various cellular processes. To gain deeper insight into the mechanisms underpinning these devastating disorders, it is essential to establish a simple and genetically tractable animal model.

**Figure 1:**
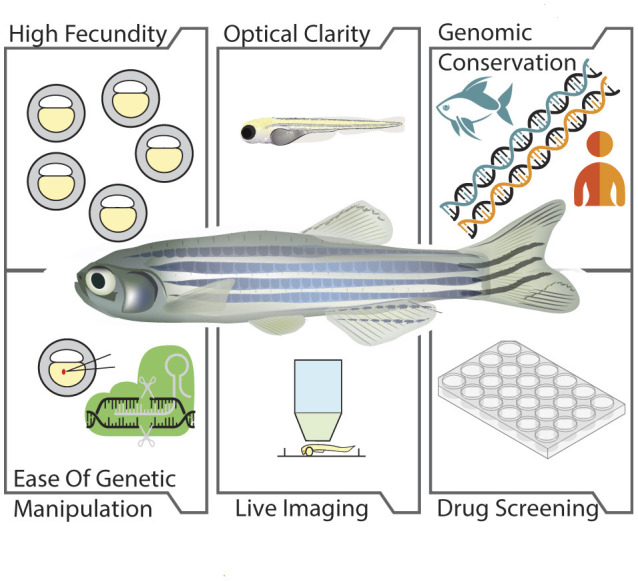
Advantages of zebrafish as a model organism. The illustration depicts the benefits of zebrafish in biological research which includes: High fecundity, capacity to produce large number of offspring; Optical clarity, transparency of the embryos and larvae; Genomic conservation, high genetic similarity with humans; Ease of genetic manipulation, ease in manipulation of risk genes associated with motor neuron diseases; Live imaging, potential for bioimaging due to optical transparency of embryos and larvae; Drug screening, ability of zebrafish for the screening of compounds with therapeutic potential.

The zebrafish, with its numerous advantages such as optical clarity of embryos, ease of genetic manipulation, rapid development, and significant genetic similarity to humans, stands out in neurological research (Figure 1). Its structural and functional parallels with mammalian neural circuitry, combined with its suitability for high-throughput drug screening, make it an invaluable tool (Laale, 1977; Lieschke and Currie, 2007). These attributes facilitate a deeper understanding of neurological disorders and accelerate the development of therapeutic strategies, bridging the gap between laboratory research and clinical applications for human patients.

This review explores how the zebrafish complements and augments the capabilities of other invertebrate and vertebrate animal models and updates existing reviews on this topic (Patten et al., 2014). We delve into innovative methods of genetic manipulation within the zebrafish genome and highlight recent strides in modeling HSP, ALS, and SMA. Additionally, we discuss the role of zebrafish in advancing drug screens, contributing to the broader spectrum of research efforts aimed at treating these and other neurological conditions.

## 2 How zebrafish stands out as a model organism?

Zebrafish were first introduced in biology research by George Streisinger in the 1970s (Streisinger et al., 1981). Later in the 1990s, Christiane Nsslein-Volhard and colleagues, employing a forward genetics approach, established zebrafish as a classical model for developmental biology and embryological studies (Driever et al., 1996; Haffter et al., 1996; Amsterdam et al., 1999). Recently, however, the scope of zebrafish research has significantly expanded. Now, it is increasingly recognized for its potential in modeling neurodegenerative disorders, especially the MNDs (Patten et al., 2014; Chia et al., 2022). The zebrafish model stands out in disease modeling due to the optical transparency of its embryos and larvae. This key feature significantly aids in the phenotypic characterization of mutations associated with MNDs, allowing researchers to observe common pathogenic features, such as curly tails, small heads and eyes, abnormal fin morphology, and motor axon outgrowth defects, with remarkable clarity (McWhorter et al., 2003; Ramesh et al., 2010; Martin et al., 2012). These morphological phenotypes are often accompanied by phenotypical behaviors and zebrafish emerged as an excellent vertebrate model for high throughput behavioral screening (Gerlai, 2010; Pelkowski et al., 2011; Green et al., 2012; Miscevic et al., 2012; Ingebretson and Masino, 2013; Richendrfer and Créton, 2013; Bruni et al., 2014; Clift et al., 2014; Garg et al., 2022). Deficits in the locomotor behavior characterize almost all types of MNDs and larval zebrafish can be elicited to exhibit their locomotor behavior through auditory, visual, olfactory, vestibular, heat, and touch stimulation (Orger and de Polavieja, 2017).

The zebrafish larvae shows burst swimming after hatching at 48–72 hpf which then changes to beat and glide swimming by 4 days post fertilization (dpf) (Saint-Amant and Drapeau, 1998; Buss and Drapeau, 2001; Drapeau et al., 2002; Brustein et al., 2003). Alternating light-dark test, in which larval zebrafish more than 4dpf, when exposed to alternating light and dark stimuli, develop a pattern of resting state in light followed by a pattern of increased movement in the dark (Burgess and Granato, 2007). This test is extensively used these days for high-throughput screening of a number of neuroactive drugs (Basnet et al., 2019). In contrast to larvae, adult zebrafish uses undulatory swimming as their principal mode of locomotion, which involves the production of a traveling wave of increasing amplitude which runs along the body down to the caudal fin that propels the fish forward (Gray, 1933; Wardle et al., 1995; Lauder and Tytell, 2005; Müller and Van Leeuwen, 2006; Smits, 2019). Generally, the amplitude of the wave motion decreases over the due course of the swimming period and next phase starts before the previous one is terminated (Müller et al., 2000). Different locomotion patterns produced by adult zebrafish including thrust, slip, yaw, saccades can be evaluated in fish with mutation in MND-associated genes for characterizing the swimming deficits (Garg et al., 2022).

Using the aforementioned stimuli, the swimming activity of zebrafish can be reliably elicited and studied with high-throughput automatic tracking methods. The tracking methods for zebrafish and other limbless organisms include several sophisticated trackers such as the Multi Worm Tracker (Swierczek et al., 2011), idTracker (Pérez-Escudero et al., 2014; Romero-Ferrero et al., 2019), and TRex (Walter and Couzin, 2021). These trackers manage uniform backgrounds and iso-illumination, simplifying the tracking process. A few tracking projects are focused on larval tracking such as Zebrafish Larvae Position Tracker (Z-LaP Tracker) (Gore et al., 2023), pi-tailtrack (Randlett, 2023), and ZebraZoom (Mirat et al., 2013). In contrast, LACE (Garg et al., 2022) focuses on adult zebrafish. Recently, more advanced image recognition trackers such as DeepLabCut, based on ResNet neural networks, have been fine-tuned on visual markers to improve accuracy in complex environments (Mathis et al., 2018). Additionally, simpler yet effective image recognition trackers like YOLO can be utilized for similar purposes (Hussain, 2023). Each of these trackers offers unique advantages depending on the specific requirements and complexities of the tracking environment.

Furthermore, the development of transgenic zebrafish enables targeted studies on the effects of mutations in specific tissues or organs (Udvadia and Linney, 2003). This contrasts with vertebrate models like mice, where observing disease phenotypes, particularly at the organ and organ system levels, generally requires invasive methods like surgery or post-mortem examination. Beyond its optical transparency, the zebrafish model boasts several other advantages: external fertilization, rapid development, high fecundity, and straightforward breeding and maintenance protocols. These attributes collectively establish zebrafish as an exemplary model system for biomedical research (Choi et al., 2021).

Advancements in sequencing technologies culminating in the successful completion of the zebrafish genome sequencing project have revealed that zebrafish share high similarity with humans at the genetic level. Nearly 70–80% of the human genes possess at least one zebrafish ortholog (Howe et al., 2013). It also includes several known and putative genes for different neurodegenerative disorders, including MNDs. Manipulating the risk genes associated with MNDs is possible in zebrafish by applying forward or reverse genetic approaches (Dosch et al., 2004; Fassier et al., 2010). Although the invertebrate and mammalian systems are also ideal for these mutagenic strategies, there are disadvantages to using both of them. Despite the high degree of functional conservation in cell biological processes, which can be modeled successfully at the genetic and molecular levels, there is lack of some essential structures e.g. innervation patterns of muscles and the myelin sheath around neurons. These differences, especially the lack of myelin, impose limitations to the efficacy of flies in studying the pathogenesis of MNDs. Conversely, mice, which share high genomic and physiological conservation with humans, are not as feasible for large scale genetic screens due to the substantial manpower, infrastructure, and financial resources required.

In light of these constraints, such as limited personnel, funding, and infrastructure, zebrafish emerge as an exceptionally advantageous model. This advantage is epitomized by the rapid development of its central nervous system (CNS), which becomes fully functional within 72 h post fertilization (hpf) (Fulwiler and Gilbert, 1991; Kimmel et al., 1995; Blader and Strähle, 2000; Schmidt et al., 2013). Complementing this rapid development, the zebrafish embryo's optical transparency during the development period allows for high-resolution live imaging of the entire CNS (Hasani et al., 2023). This capability not only facilitates detailed observation but also underscores a significant aspect of zebrafish research: the striking similarity between the anatomical structure and function of the zebrafish and mammalian brains (Fulwiler and Gilbert, 1991; Blader and Strähle, 2000; Howe et al., 2013; Schmidt et al., 2013). This resemblance extends to neural circuitry and neuroendocrine functions, with a notable conservation of the neurotransmitter system across species. Key neurotransmitters, including serotonin (5-HT), dopamine (DA), histamine (HA), acetylcholine (ACh), glutamate and gamma-aminobutyric acid (GABA) are synthesized similarly in both zebrafish and mammals (Kaslin and Panula, 2001; Panula et al., 2006; Wasel and Freeman, 2020). Despite these advantages, neuroscientists often express concern regarding the use of zebrafish for modeling MNDs, primarily due to the absence of corticospinal and rubrospinal tract in this model organism. This absence potentially limits the zebrafish's capacity to replicate the clinical phenotypes of the patients (Babin et al., 2014). Furthermore, there are organizational and functional differences in the motor neurons of zebrafish and humans. Human motor neurons in the spinal cord are organized in specific motor columns along a medial-lateral axis, targeting different muscle groups for movement (Miall, 2022). This precise topography is crucial for the coordinated control of complex motor functions and is a characteristic shared with other mammals but not with more basal vertebrates (Fetcho, 1992). In contrast, spinal motor neurons in zebrafish are categorized into primary motor neurons (PMNs) and secondary motor neurons (SMNs) based on their timing and target musculature. SMNs have smaller somatas, thinner axons, and they are localized more ventrally in the motor column, functioning to regulate the speed of rhythmic swimming (Myers, 1985; Myers et al., 1986). Contrarily, PMNs which control escape movement are further divided into three sub-types, caudal, middle and rostral primary motor neurons, based on the soma position and specific axonal pathways within the spinal cord (Myers et al., 1986; Westerfield et al., 1986). While the PMNs fire action potentials at ultrahigh frequency, SMNs do so at lower frequency and are prone to frequent failure (Wang and Brehm, 2017; Wen et al., 2020). However, an intriguing aspect of zebrafish neuroanatomy offers a potential bridge to this gap: the presence of Mauthner cells in their hindbrain. Mauthner cells, the fastest motor neurons known, govern the rapid escape reflex called the C-start response (Eaton et al., 1977; Faber and Korn, 1978). Intriguingly, these cells maintain projections to the lumbar spinal cord in adult amphibians even after metamorphosis (Will, 1986; Davis and Farel, 1990). These results signify that Mauthner cells could have a similar role in adult amphibians and thus could serve as a functional reminiscent of the corticospinal tract. Ongoing research aimed at unraveling the role of Mauthner cells in zebrafish and adult amphibians could open new avenues for using zebrafish more effectively in modeling neurodegenerative diseases, especially the MNDs. Leveraging the structural and functional conservation of the zebrafish and mammalian system, researchers developed numerous zebrafish models for genetic disorders including neurodegenerative disorders (Naef et al., 2019; Chia et al., 2022). These models, generated through labor and cost-effective genetic screens, are instrumental in advancing our understanding of such disorder.

A key advantage of using zebrafish lies in its efficacy for high-throughput, inexpensive drug screening. In the last 20-25 years, zebrafish has emerged as a valuable model for understanding disease mechanisms and bridging the gap between *in-vitro* assays and mammalian drug screening (Lieschke and Currie, 2007; Patten et al., 2014). Zebrafish have consistently demonstrated that compounds effective in human and mouse systems exhibit similar activity and pharmacokinetic properties in zebrafish (MacRae and Peterson, 2015). Zebrafish has been widely used for both *in-vitro* and *in-vivo* drug screening. While the *in-vitro*, which focuses on the disruption of validated targets, works well in the early stages, they often fall short at advanced levels. The major drawback of such target-based approaches is that they usually modify the molecular target rather than the disease phenotype. Conversely, *in-vivo* in zebrafish is to identify the compounds that alter the disease phenotype on an organismic level, rather than solely focusing on a specific molecular target (Zon and Peterson, 2005). Despite discovering numerous new compounds from drug screens in zebrafish, the pivotal question remains: how effectively will these drugs translate to human treatments? Preliminary findings suggest a high level of conservation in drug responses between zebrafish and humans. While mammalian models remain the gold standard for testing the efficacy of drugs, particularly in late stages, zebrafish studies offer a rapid and cost-effective method for the preliminary evaluation of compounds with therapeutic potential (Patton et al., 2021).

## 3 Zebrafish toolbox for genetic manipulation

Optical transparency of embryos and larvae during development and a high degree of synteny of the zebrafish genes to the human sequences makes zebrafish a particularly amenable model for studying a variety of human genetic disorders, including MNDs. The methods available to make genetic manipulations in the zebrafish genome are broadly classified into three categories:

### 3.1 Forward genetic methods

**Figure 2:**
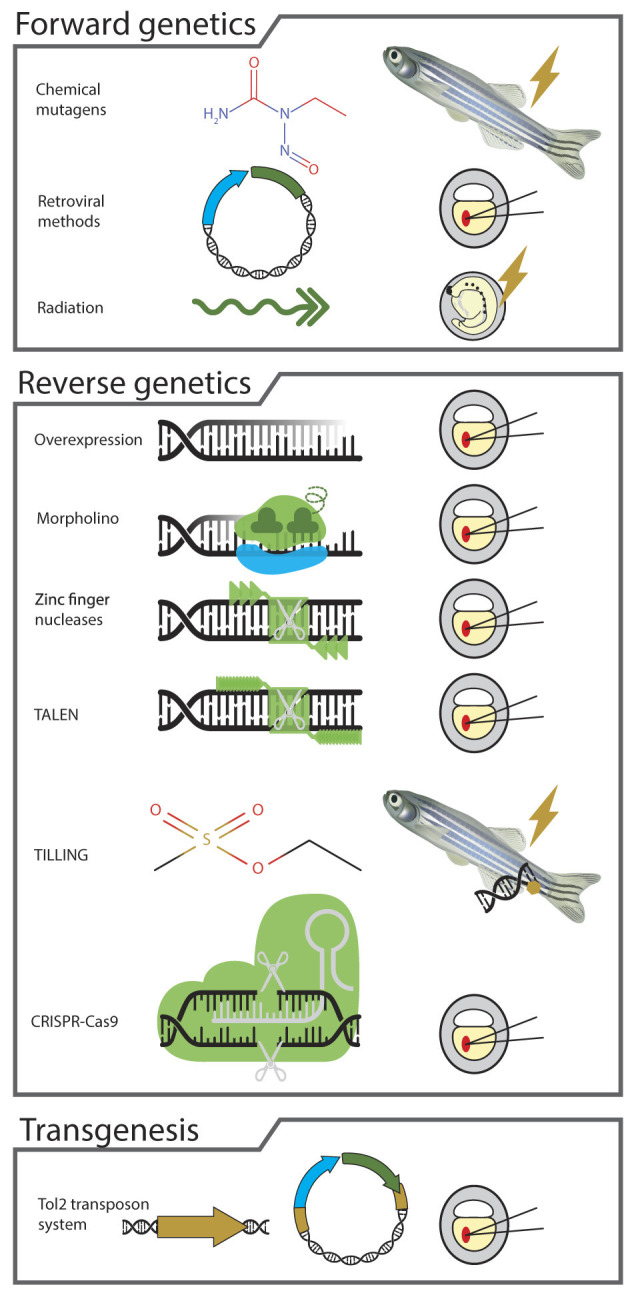
The diagram highlights different methods available for the genetic manipulation of zebrafish genome. The forward genetic approaches includes mutagenesis via chemicals such as N-ethyl-N-nitrosourea (ENU), retroviral methods and radiation. The reverse genetic methods include overexpression, injection of morpholinos, zinc finger nucleases and transcription activator like effectors (TALEN). It also includes mutagenesis via targeting induced local lesions in genomes (TILLING) and clustered regularly interspaced short palindromic repeats-crispr associated protein 9 (CRISPR-Cas9). Further, the illustration shows that transgenesis via Tol2 transposon system is also a method often used to manipulate the zebrafish genome for understanding the role of the risk genes associated with motor neuron and other diseases.

Forward genetics is a phenotype-based approach used for determining the genetic basis of a particular phenotype or trait of interest (Driever et al., 1996; Haffter et al., 1996). The phenotype is caused by random mutagenesis through chemicals, radiations or retroviral methods (Figure 2). To generate mutants with the chemical method, male fish are exposed to mutagen N-ethyl-N-nitrosourea (ENU), which induces point mutations in the coding or non-coding regions of the genome. Thanks to their notable resistance to mutagen toxicity, zebrafish enable the targeted mutation of specific loci, a feat that proves challenging in other vertebrate models (Driever et al., 1996; Haffter et al., 1996; Wienholds et al., 2002; Dosch et al., 2004). Additionally, mutagenesis using retroviral methods has shown considerable success in zebrafish. This approach circumvents the labor-intensive process of positional cloning often necessary in forward genetic screens (Amsterdam et al., 1999, 2004). Among the various techniques for forward genetic screens, the use of radiation is the least explored. Nevertheless, studies have demonstrated the efficacy of gamma rays as a mutagen, striking specific loci with a rate of approximately 1:100, making it a potent tool in zebrafish genetic research (Chakrabarti et al., 1983; Walker and Streisinger, 1983; Lu et al., 2007). The crux of the forward genetic approach is its ability to generate mutants with distinct phenotypes, easily observable in the transparent embryos and larvae of zebrafish without the need for advanced instruments and facilities. This method is particularly effective for creating mutations in genes that are orthologous to human genes implicated in the pathogenesis of genetic disorders, including MNDs. For instance, Dosch et al. (2004) employed a maternal effect mutant screen using ENU in zebrafish, leading to the identification of four mutants: *ruehrei^p25ca^*, *over easy*^p37ad^, *sunny side up^p144dc^*, *souffle^p96re^*, all exhibiting a specific opaque egg phenotype. These mutants resemble the appearance of immature stage IV oocytes, pointing to a potential defect in oogenesis (Dosch et al., 2004).

Interestingly, Kanagaraj et al. (2014) established that *souffle* is the zebrafish ortholog of the HSP-associated gene *ZFYVE26*, which codes for the protein SPASTIZIN. The *souffle* mutants possess a single-point mutation in the splice donor site, resulting in a frameshift and production of a shortened 2,270 amino acid Spastizin protein. Notably, while larvae and adult *souffle* mutants exhibit very mild locomotion defects, a more severe mutation in *zfyve26* results in mutants that not only share the opaque egg phenotype but also display slower swimming compared to their wild-type counterparts. These forward genetic screens are immensely beneficial, as any mutants displaying similar phenotype, are immediately highlighted as the potential disease model, in this case, for HSP. Once validated as models for HSP, these mutants offer valuable insights into the molecular and cellular basis of the pathogenesis of this complex disorder.

### 3.2 Reverse genetic methods

Unlike forward genetics, which explores the genetic basis of a phenotype, reverse genetics starts with a known gene and investigates its function by analyzing the phenotypic effects of specific genetic manipulations. In zebrafish research, several methods are available for modifying the zebrafish orthologs of human disease genes (Figure 2). One of the simplest strategies for investigating gene function in the optically transparent embryos and larvae involves either overexpression using mRNA or knockdown by antisense morpholino oligonucleotides (MOs). Both methods have been instrumental in investigating genes affected in MNDs. For example, Fassier et al. (2010) employed both methods to explore the role of Atlastin-1 in the pathogenesis of HSP. Their findings revealed that the knockdown of *atlastin-1* resulted in a significant reduction in larval motility, increased branching of spinal motor axons, and marked upregulation of bone morphogenetic protein (BMP) signaling pathway. Conversely, overexpression of *atlastin-1* inhibited the BMP signaling. However, major limitations of the MO injection include incomplete knockdown, off-target deleterious effects and transient expression, which may not manifest in juvenile and adult stages.

Advancements in technologies like zinc finger nucleases (ZFNs), transcription activator-like effectors (TALENs), and targeting-induced local lesions in genomes (TILLING) have significantly enhanced the precision and efficiency of targeted mutagenesis and gene function ablation in zebrafish. ZFNs function by coupling a zinc finger DNA-binding domain with a DNA cleavage domain, specifically developed to target and cleave precise DNA sequences within the genome (Ekker, 2008). TALENs operate through a similar mechanism, where transcription activator-like (TAL) effector DNA binding domains are fused to DNA cleavage domains, functioning as bespoke restriction enzymes (Huang et al., 2016). TILLING, another popular method among zebrafish researchers, merges chemical mutagenesis with sensitive DNA screening, effectively pinpointing point mutations in targeted genes (Moens et al., 2008). Although all these methods have come up as efficient alternatives against MO injections, they have their own limitations as they are complex and costly. Until now, clustered regularly interspaced short palindromic repeats-CRISPR associated protein 9 (CRISPR-Cas9) has turned out to be the most efficient technique for targeted gene knockout in zebrafish (Hwang et al., 2013; Irion et al., 2014; Li et al., 2016; Liu et al., 2019a; Uribe-Salazar et al., 2022). This method is substantially used for producing both knockout and knock-in mutants. It comprises two key components: guide RNA and Cas9. The guide RNA is designed to match the desired target gene, while Cas9 induces a double-stranded DNA break. Together, these elements facilitate precise genome modification at a desired destination. Previous studies have reported the issue of off-target toxicity while using this method. Still, great effort has been put in recently to improve the specificity of Cas9 and to broaden the target coverage of the CRISPR nucleases (Liu et al., 2019a).

Several zebrafish models of MNDs have been developed recently using the CRISPR-Cas9 system. Armstrong et al. (2016) modeled ALS in zebrafish by creating site-specific single nucleotide mutations for the first time in two ALS-related genes *tardbp* and *fus* using CRISPR-Cas9 mediated homology-directed repair by co-injection of single-stranded oligodeoxynucleotide donor templates (ssODN) with gRNA and Cas9 mRNA. In another study, Voisard et al. (2022) developed a stable constitutive *valosin-containing protein (vcp)* knockout zebrafish model and showed that loss of *vcp* affects protein homoestasis followed by the impairment of cardiac and skeletal muscle function. In addition, zebrafish models exhibiting a loss of function for *tumor protein 73 (tp73)* and *transactive response DNA binding protein of 43 kDa (tdp-43)* have been developing using CRISPR-Cas9 (Bose et al., 2019a; Russell et al., 2021). These models display hallmark features of ALS pathology including disturbances in motor neuron development and abnormalities in axon morphology.

### 3.3 Transgenic methods

Diverging from both forward and reverse genetics, transgenesis in zebrafish enables the targeted expression of human disease genes, offering precise control over their spatial and temporal dynamics in the study of motor neuron diseases. Transgenesis stands as a distinct approach in zebrafish models for the detailed exploration of human motor neuron diseases (Balciunas et al., 2006). This technique hinges on the introduction of human disease genes into zebrafish, a process efficiently executed through Tol2 transposon system (Figure 2). Injection of Tol2 constructs along with promoters for neural-specific expression in the fertilized oocytes leads to the generation of zebrafish models expressing disease-causing genes in desired parts of the nervous system (Kawakami, 2004; Suster et al., 2009). Transgenesis via the Gal4-UAS is another effective method for observing the effects of human mutations in the zebrafish system (Asakawa and Kawakami, 2008; Halpern et al., 2008). Both methods help control the timing, location and quantity of disease gene expression, thereby avoiding the possibility of lethality due to ectopic or toxic overexpression. Hao et al. (2011) generated transgenic zebrafish expressing human survival motor neuron *(hSMN2)* gene whose low levels cause SMA. They showed that the splicing mechanism for SMN2 is conserved in zebrafish, like in mice and humans. Furthermore, their research revealed that injecting antisense morpholinos (MOs) targeting the intronic splicing silencer site resulted in a notable elevation in the levels of full-length SMN. When introduced into an *smn* mutant background, this increase in SMN successfully ameliorated the neuromuscular defect associated with the presynaptic synaptic vesicle protein 2 (SV2). Notably, it prolonged the survival of the mutant zebrafish.

## 4 Hereditary spastic paraplegia

Hereditary Spastic Paraplegias (HSPs) are a clinically diverse group of inheritable neurodegenerative disorders in which people suffer from lower limb spasticity and weakness, resulting from the length-dependent degeneration of upper motor neurons in the corticospinal tract (Harding, 1984, 1993; Fink, 2006, 2014; Klebe et al., 2015; Tesson et al., 2015). With over 88 genes and 100 distinct spastic gait disease loci identified as causative factors for Hereditary Spastic Paraplegia (HSP) (Galatolo et al., 2018; Parodi et al., 2018; Elsayed et al., 2021), patients continue to face a dire situation due to the lack of a definitive cure. However, some therapeutics are available to alleviate the pathology in human patients. Interestingly, HSP genes are involved in several cellular processes, including endoplasmic reticulum (ER) morphogenesis, endosomal trafficking, lipid metabolism, mitochondrial regulation, myelination, autophagy, etc. (Blackstone, 2012, 2018). Despite the diverse cellular processes implicated, the inheritance of HSP genes is equally varied, encompassing autosomal dominant, autosomal recessive, X-linked and maternal patterns (Finsterer et al., 2012). Nearly 10–40% cases of the autosomal dominant form of HSP are due to mutations in *ATLASTIN-1* and *SPASTIN*, respectively. Atlastin-1 is a GTPase required for ER morphogenesis and BMP signaling. Moreover, it is involved in vesicle trafficking and neurite outgrowth (Liu et al., 2019b). Conversely, Spastin is a microtubule severing AAA ATPase protein which also plays a role in ER morphogenesis, BMP signaling, endosomal trafficking, and cytokinesis (Hazan et al., 1999; Errico et al., 2002). In addition, Spastin is furthermore required for motor axon outgrowth. Interestingly, Atlastin-1 and Spastin also interact with each other and coordinate microtubule interactions with the tubular ER network (Evans et al., 2006; Sanderson et al., 2006). The two most common forms of autosomal recessive HSP are SPG11 and SPG15, caused by mutations in *SPATACSIN* and *SPASTIZIN*, respectively. Spatacsin and Spastizin share similarities in their expression patterns and interact with each other and play a pivotal role in endosomal trafficking and the generation of new lysosomes (Chang et al., 2014). Mutations in *MCT8* and *L1CAM* are associated with the X-linked form of HSP. Monocarboxylate transporter 8 (MCT8) is a transporter of a variety of iodothyronine, including thyroid hormones T3 and T4 (Friesema et al., 2003). L1 cell adhesion molecule (L1CAM) is a transmembrane protein implicated in cell adhesion, migration, myelination, neurite extension and differentiation (Kiefel et al., 2012).

Zebrafish have risen to prominence as a valuable model for simulating HSP research with over 40 studies published to date (Naef et al., 2019). Despite the absence of a corticospinal tract, limiting its ability to fully mimic HSP pathology in humans, zebrafish models have successfully exhibited key pathological features of HSP patients, such as impaired locomotion, disrupted motor axon growth, and axonal transport issues (Table 1). For instance, *atl1* mutation in zebrafish results in markedly reduced larval locomotion, increased branching of spinal motor axons, and upregulation of the BMP signaling pathway; its overexpression leads to inhibition of the BMP signaling (Fassier et al., 2010). Similarly, *spastin* morphants also possess locomotion defects along with a curly tail phenotype, shorter and disordered axons, defects in motor axon outgrowth, and disorganization and thinning of microtubule networks in the spinal cord (Wood et al., 2006; Julien et al., 2016). Furthermore, targeting two Spastin isoforms DrM1 and DrM61 leads to reduced swimming speed and distinct phenotypic changes such as curly tails in *DrM1* mutants and smaller eyes and yolk tube agenesis in *DrM61* mutants, with embryos from both failing to hatch (Jardin et al., 2018).

**Table 1:** Characterisation of the disease phenotypes in zebrafish model of HSP.

**Human genes**	**Zebrafish genes**	**Zebrafish modeling**	**Motor deficit**	**Other phenotype**	**References**
*ATL1*	*atl1*	Morphant	Reduced larval locomotion	Increased branching of spinal motor axons and upregulation of BMP signaling	Fassier et al., 2010
	*atl1*	Overexpression	-	Inhibition of BMP signaling	Fassier et al., 2010
*SPAST*	*spast*	Morphants	Locomotion defects	Curly tail, defects in motor axon outgrowth, thinning of spinal cord microtubule networks	Wood et al., 2006; Julien et al., 2016
	*spast*	DrM1 Morphants	Locomotion defects with reduction in swimming speed	Embryos fail to hatch from chorion, curly tail	Jardin et al., 2018
	*spast*	DrM61 Morphants	Locomotion defects with reduction in swimming speed	Embryos fail to hatch from chorion, small eyes, yolk tube agenesis	Jardin et al., 2018
*KIF5A*	*kif5A*	Mutant	Reduced touch response, sensory and motor deficits	Inflated swim bladder, larval lethality	Campbell et al., 2014; Auer et al., 2015
*WASHC5*	*strump- ellin*	Morphant	Impaired motility	Curly tail, shortening and abnormal branching of motor and interneurons, loss of motor neuron formation, pericardial edema, extended heart cavity	Valdmanis et al., 2007; Clemen et al., 2010
*BSCL2*	*seipin*	Mutant	Reduced swimming activity	Reduction in d triglyceride in developing head, no morphological deformities or motor neuron loss	Hölttä-Vuori et al., 2013
*SLC33A1*	*slc33a1*	Morphant	-	Curly tail, defects in the organization of motor axons and axon outgrowth	Lin et al., 2008; Mao et al., 2015
*UBAP1*	*ubap1*	Mutant	Decreased movement	Truncated and misshaped motor axons, morphological changes, shorter life span	Fard et al., 2019; Lin et al., 2019
*ALSIN*	*alsin*	Morphant	Behavioral and swimming defects	Abnormalities in motor neuron outgrowth	Gros-Louis et al., 2008
*SPATACSIN*	*spatacsin*	Morphant	Reduced motility or paralysis	Abnormalities in motor neuron axon outgrowth and CNS, other developmental defects	Southgate et al., 2010; Martin et al., 2012; Boutry et al., 2018
*ZFYVE26 SPASTIZIN*	*zfyve26 spastizin*	Morphant, Mutant	Motor deficits	Motor axon outgrowth defects, defects in secretory vesicle maturation during oogenesis	Martin et al., 2012; Kanagaraj et al., 2014
*PNPLA6*	*PNPLA6*	Morphant	-	Developmental defects, curly tail, small head and aberrant eyes, defective axonal transport, less motor neurons	Song et al., 2013; Hufnagel et al., 2015
*GBA2*	*gba2*	Morphant	Locomotor abnormalities	Curly tail, impairment in axonal outgrowth	Martin et al., 2013
*AP4S1*	*ap4s1*	Morphant, Mutant	Motor impairment	Abnormal CNS development and neuronal excitability, delay in neurodevelopment and distal axon degeneration	DAmore et al., 2020; Li et al., 2023
*VPS37A*	*vps37a*	Morphant	Severe loss of motility	-	Zivony-Elboum et al., 2012
*CAPN1*	*capn1*	Morphant	-	Disorganized axonal network, aberrant branchiomotor neuron migration	Gan-Or et al., 2016
*KLC2*	*klc2*	Morphant	-	Curly tail phenotype, high mortality	Melo et al., 2015
	*klc2*	Overexpression	-	Curly tail phenotype, high mortality	Melo et al., 2015
*PCYT2*	*pcyt2*	Mutant *pcyt2_3*	-	Larval lethality	Vaz et al., 2019
*PCYT2*	*pcyt2*	Mutant *pcyt2_13*	-	Small size, abnormal tail fin morphology	Vaz et al., 2019
*AMFR*	*amfr*	Mutant	Aberrant touch response	Shorter in length, lipid accumulation, aberrant ER morphology, abnormal motor neuron branching	Deng et al., 2023
*RNF170*	*rnf170*	Morphant	Loss of motility	Microphthalmia, Microcephaly	Wagner et al., 2019
*HPDL*	*hpdl*	Morphant	Motor impairment	-	Wiessner et al., 2021
*COQ4*	*coq4*	Morphant	Severe motor dysfunction	-	Mero et al., 2021; Lin et al., 2023
*MCT8*	*mct8*	Mutant	Motility defects, decrease in response to external stimuli	Defects in neural circuit assembly, disturbances in myelination	Zada et al., 2014, 2016
*L1CAM*	*l1.1*	Morphant	-	Defective axonal outgrowth and myelination and development of hydrocephalus	Linneberg et al., 2019

Mutations in kinesin family member 5A *(KIF5A)*, which plays a crucial role in the axonal transport of cargos, cause SPG10 in nearly 3% of families. Mutations in *kif5a* in zebrafish lead to larval lethality, reduced touch response, inflated swim bladder, and sensory and motor deficits (Campbell et al., 2014; Auer et al., 2015). Another autosomal dominant form of HSP, SPG8, is caused by mutations in *STRUMPELLIN* which is involved in endosomal trafficking. Zebrafish *strumpellin* morphants possess impaired motility, curly tails, extended heart cavity, pericardial edema, and either shorter spinal cord motor and interneurons with abnormal branching or a loss of central and peripheral motorneuron formation (Valdmanis et al., 2007; Clemen et al., 2010).

SEIPIN, an ER protein involved in lipid metabolism, is another protein involved in HSP pathogenesis. Although *seipin* mutant zebrafish lack any morphological deformities or motor neuron loss, they exhibit indeed slower swimming. *seipin* mutants possess reduced triglyceride content in the developing head (Hölttä-Vuori et al., 2013). Similar phenotypes are observed with knockdowns of other ER proteins, such as acetyl-CoA transporters, leading to curved tails and axon outgrowth anomalies. A newly identified autosomal dominant HSP gene is ubiquitin-associated protein 1 *(UBAP1)*. A truncating mutation in *ubap1* resulted in truncated and misshaped axons in the fluorescently labeled motor neurons of transgenic Tg*(olig2::DsRed)* zebrafish embryos at 48 hpf (Fard et al., 2019). In another recent study, knockdown of *ubap1* in zebrafish leads to abnormalities in morphology, decreased movement, shorter life span and inhibition of motor neuron outgrowth (Lin et al., 2019).

Studies on zebrafish have extensively explored the effects of mutations in genes associated with autosomal recessive forms of HSP. Notably, mutations in *alsin*, integral for vesicle trafficking in neurons, result in developmental and behavioral defects in zebrafish embryos (Gros-Louis et al., 2008). MO-mediated knockdown of *spatacsin* in zebrafish leads to a range of abnormalities including reduced motility or paralysis, aberrant spinal cord motor neuron axon outgrowth, other developmental defects and central nervous system (CNS) irregularities (Martin et al., 2012). In the same study, Martin et al. (2012) also targeted *spastizin* with the MO and observed similar phenotypes in *spastizin* morphants just like in *spatacsin* ones.

Intriguingly, in another study in zebrafish, Kanagaraj et al. (2014) found that while zygotic *spastizin* mutants do not possess any defects in locomotion and axonal outgrowth, they possess defects in secretory vesicle maturation during oogenesis. Other less common types of autosomal recessive HSP include SPG39, SPG46, SPG52, SPG53, SPG76, as well as mutations in kinesin light chain 2 *(KLC2)*, phosphate cytidylyltransferase 2, ethanolamine *(PCYT2)*, autocrine motility factor receptor (AMFR), ubiquitin E3 ligase *RNF170*, Human 4-hydroxyphenylpyruvate dioxygenase-like (HPDL), and Coenzyme Q4 (COQ4) have also been identified. SPG39 arising from mutations in patatin-like phospholipase domain containing 6 *(PNPLA6)* leads to developmental abnormalities and axonal transport issues in zebrafish morphants (Song et al., 2013; Hufnagel et al., 2015). Mutation in SPG46 gene glucosylceramidase β2 *(GBA2)* involved in lipid metabolism, results in abnormal locomotion and impaired axonal outgrowth in zebrafish morphants (Martin et al., 2013). SPG52 is caused due to mutations in AP4S1, σ4 subunit of the adaptor protein complex 4 (AP-4). MO-mediated knockdown of *ap4s1* in zebrafish leads to abnormalities in CNS development and neuronal excitability followed by locomotion defects (DAmore et al., 2020). CRISPR induced mutation in *ap4s1* also causes motor impairment as well as delay in neurodevelopment and degeneration of distal axons (Li et al., 2023). SPG53 is caused by mutations in vacuolar protein sorting 37 homolog A *(VPS37A)*. VPS37A is part of the endosomal sorting complex required for the transport (ESCRT) system, which is involved in intracellular trafficking, protein sorting, and maturation of multivesicular bodies. Mutation in *vps37a* causes severe loss of motility in zebrafish morphant embryos (Zivony-Elboum et al., 2012). Furthermore, SPG76 is caused by a mutation in *CAPN1*, which encodes CALPAIN-1. CALPAIN-1 is widely expressed in CNS and essential for the maintenance of axonal and synaptic plasticity. Injection of *capn1a-mo* in a transgenic zebrafish line Tg*(islet1::GFP)* expressing the green fluorescent protein (GFP) in motor neurons resulted in abnormalities in the organization of the axonal network and branchiomotor neuron migration (Gan-Or et al., 2016). *KLC2* mutations also serves as a cause for autosomal recessive form of HSP. Effects of both knockdown and overexpression of the gene have been studied in zebrafish, and mutants showed mild to severe curly tail phenotype (Melo et al., 2015). Mutations in genes governing lipid metabolism also cause autosomal recessive HSP. *PCYT2* encodes an enzyme for phosphatidylethanolamine synthesis, a major membrane lipid in the brain. *pcyt2* knockout zebrafish, created by targeting exon 3 showed higher mortality compared to the one created by targeting the final exon 13. The *pcyt2_13* mutants had a higher survival rate but possessed an overall small size and abnormal tail fin morphology (Vaz et al., 2019). AMFR codes for a RING-H2 finger E3 ubiquitin ligase which is anchored at the ER membrane. *amfr* zebrafish mutants are shorter in length, exhibit aberrant ER morphology and lipid accumulation in brain, followed by abnormal motor neuron branching and aberrant touch evoked escape response (Deng et al., 2023). Interestingly, MO-mediated knockdown of another E3 ubiquitin ligase gene *rnf170* in zebrafish causes loss of motility and developmental defects including microphthalmia, microcephaly (Wagner et al., 2019). *hpdl* is an iron-containing non-heme oxygenase, localized to the outer mitochondrial membrane, whose knockdown resulted in abnormal locomotor behavior in zebrafish larvae (Wiessner et al., 2021). Using “crispant” approach, Mero et al. (2021) showed that *coq4* mutant fish exhibit motor defects and Purkinje cell reduction in a specific hindbrain area, which is a reminiscent of the human cerebellum. These findings were supported by a very recent study in which Lin et al. (2023) also showed that knockdown of *coq4* leads to severe motor dysfunction in zebrafish.

Mutations in *mct8* are associated with defects in neural circuit assembly, reduced locomotion and anomalies in the expression of myelin-related genes in zebrafish. Subsequent analysis of the *mct8* mutants revealed disturbances in the myelination process, characterized by increased Schwann cells in the trunk and a concurrent decrease of oligodendrocytes in the brain and spinal cord (Zada et al., 2014, 2016). Similarly, knockdown of *l1cam* in zebrafish caused myelination abnormalities, changes in axonal outgrowth and development of hydrocephalus (Linneberg et al., 2019).

In conclusion, zebrafish models have been instrumental in advancing our understanding of Hereditary Spastic Paraplegia (HSP), a group of neurodegenerative disorders characterized by lower limb spasticity and weakness. Despite the absence of a corticospinal tract, these models have successfully mimicked key pathological features of HSP, including impaired locomotion and motor axon outgrowth. Research has identified mutations in various genes, such as *ATLASTIN-1, SPASTIN, SPASTIZIN*, and *SPATACSIN*, and their diverse roles in cellular processes like ER morphogenesis, endosomal trafficking, and lipid metabolism, contributing to HSP pathogenesis. Future research should focus on deeper molecular and cellular studies, especially on how these genetic mutations disrupt specific neuronal pathways. Developing targeted gene therapies and further exploring the role of these genes in motor neuron biology could pave the way for new treatment strategies. Additionally, the creation of more advanced zebrafish models that can better mimic the human condition of HSP would greatly contribute to understanding the disease's progression and testing potential therapeutics.

## 5 Amyotrophic lateral sclerosis

Amyotrophic Lateral Sclerosis (ALS), commonly known as Lou Gehrig's disease is a neurodegenerative disorder, characterized by the progressive loss of the upper and lower motor neurons (Kiernan et al., 2011; Brown and Al-Chalabi, 2017). This deterioration leads to muscle weakness, atrophy, spasticity, followed by paralysis and eventually death due to respiratory failure. The disease's incidence stands at approximately 2–3 per 10,000 individuals (Loeffler et al., 2016; Grad et al., 2017; Matsubara et al., 2019). ALS is broadly classified into two categories: sporadic and familial. The sporadic form accounts for about 90% of ALS cases, with the remaining 10% being familial (Kiernan et al., 2011; Brown and Al-Chalabi, 2017). Intriguingly, despite the identification of over 40 genes linked to ALS pathogenesis, mutations in just four genes–superoxide dismutase 1 (*SOD1*), *TARDBP* (which encodes the TAR DNA-binding protein-43 [TDP-43]), fused in sarcoma (*FUS*), and chromosome 9 open reading frame 72 (*C9ORF72*)–are responsible for approximately 70% of familial ALS cases (Al-Chalabi et al., 2017; Roggenbuck et al., 2017).

Superoxide dismutase 1 (*SOD1*), a widely expressed anti-oxidizing enzyme, is implicated in nearly 20% of familial and 1.7% sporadic ALS cases. Identified in the early 1990s as the first ALS-related gene and extensively studied through numerous mouse models, the exact mechanism by which *SOD1* mutations trigger motor neuron degeneration remains elusive. Evidences so far suggest that a toxic gain-of-function mechanism might be responsible for the pathogenesis of ALS due to mutations in *SOD1* (Peggion et al., 2022). Another gene, *TARDBP* encodes a nuclear DNA- and RNA-binding protein called TDP-43, a protein integral to RNA processing and metabolism, operating both at the nuclear DNA and RNA levels. Mutations in *TARDBP*, leading to an accumulation of TDP-43 in inclusion bodies, account for nearly 3% of familial and 1.5% of sporadic ALS cases (Berning and Walker, 2019). Similarly, Fused in Sarcoma (*FUS*), another nuclear DNA- and RNA-binding protein involved in RNA processing, including transcription and splicing, plays a significant role in ALS. Mutations in *FUS* lead to its mislocalisation from nucleus to the cytoplasm, which causes its aggregation in the inclusion bodies (Lattante et al., 2013). A prominent genetic factor is the extensive expansion of GGGGCC hexanucleotide repeats in the non-coding first intron of *C9ORF72*, accounting for nearly 40% of familial and 7% of sporadic ALS cases. While repeat expansions are the primary cause of motor neuron degeneration in *C9ORF72*-related ALS, the loss of function due to haploinsufficiency of *C9ORF72* is also suggested to contribute significantly to the disease's progression (Stepto et al., 2014; Balendra and Isaacs, 2018).

The seminal study examining the effects of *sod1* mutations in zebrafish was conducted 16 years ago by Lemmens et al. (2007). They discovered that mutant human *SOD1* overexpression leads to specific and dose-dependent motor axonopathy in zebrafish embryos. Subsequent research demonstrated similar neurodegenerative effects (Table 2). For instance, transient overexpression of G93A *SOD1* mutation in zebrafish caused degeneration of neuromuscular junctions (NMJs) and defects in motor neuron outgrowth and axonal branching (Sakowski et al., 2012). Robinson et al. (2019) observed analogous effects, including spinal motor axon shortening and atypical branching, upon overexpression of mutated human *SOD1* in zebrafish larvae. Notably, a novel *sod1* T70I zebrafish model developed using ENU mutagenesis and TILLING exhibits motor pathology symptoms, including motor neuron loss, susceptibility to oxidative stress, altered NMJ morphology and motor impairment in adult fish (Da Costa et al., 2014). These motor pathologies, initially observed in transient mutant human *SOD1* overexpression models, were further validated in stable transgenic zebrafish lines. Ramesh et al. (2010) developed two G93R *sod1* mutant lines which displayed NMJ defects at larval and adult stages, decreased swimming endurance during adulthood, and muscle atrophy, motor neuron loss at the end stage, ultimately leading to premature death. Benedetti et al. (2016) also reported similar pathological features in larval and adult zebrafish through stable expression of G93R mutation. Moreover, Sakowski et al. (2012) developed a stable transgenic zebrafish line expressing G93A-*sod1* mutation and observed common pathologies such as loss of NMJs, motor neuron degeneration, reduced activity, and altered motor behavior. To investigate the correlation between motor axonopathy and locomotion defects in the *sod1* mutant fish, Robinson et al. (2019) injected transgenic zebrafish embryos expressing blue fluorescent protein (mTagBFP) in motor neurons with mutant Sod1 mRNA. Larvae injected with mutant Sod1 mRNA possessed significantly shorter and more aberrantly branched motor axons. Additionally, these larvae could only swim short distances compared to controls. This observation links genetic mutations directly to motor function impairments.

**Table 2:** Delineation of the disease phenotypes in zebrafish model of ALS.

**Human genes**	**Zebrafish genes**	**Zebrafish modeling**	**Motor deficit**	**Other phenotype**	**References**
*SOD1*	*sod1*	Overexpression (G93A, G37R, A4V)	-	Specific and dose dependant motor axonopathy	Lemmens et al., 2007
	*sod1*	Overexpression (G93A)	-	NMJ abnormalities, defective axonal branching and motor neuron outgrowth	Sakowski et al., 2012
	*sod1*	Overexpression	-	Shortening of spinal motor axons and aberrant branching	Robinson et al., 2019
	*sod1*	Overexpression (T70I)	Reduction in swimming duration and velocity (in adults)	Motor neuron loss, altered NMJ morphology, susceptibility to oxidative stress	Da Costa et al., 2014
	*sod1*	Stable mutant (G93A)	Decreased swimming endurance (in adults)	Motor neuron loss, defects at NMJs, muscle atrophy, premature death	Ramesh et al., 2010; Benedetti et al., 2016
	*sod1*	Stable transgenic mutant (G93A)	Decreased activity and motor behavior	Loss of NMJs and motor neurons	Sakowski et al., 2012
*TARDBP*	*tardbp*	Morphant	Impairment of tail coiling and touch evoked escape response	Reduced axon length and abnormal branching	Kabashi et al., 2010; Demy et al., 2020
	*tardbp*	Overexpression (A315T, A382T, and G348C)	Impairment of tail coiling and touch evoked escape response	Motor neuron loss, reduced axon length and abnormal branching	Kabashi et al., 2010
	*tardbp*	Overexpression (G348C)	Impairment of swimming activity	NMJ abnormalities	Armstrong and Drapeau, 2013a; Lissouba et al., 2018
	*tardbp*	Double mutant	-	Impairment of spinal motor axon outgrowth, muscle degeneration, vasculature mispatterning, early death	Hewamadduma et al., 2013; Schmid et al., 2013; Bose et al., 2019a
	*tardbp*	CRISPR-Cas9 mediated double mutant	Locomotor defects	Morphological abnormalities, synaptic dysfunction, NMJ abnormalities	Bose et al., 2019a
*FUS*	*fus*	Overexpression (P525L)	-	Inhibition of nuclear import, aggregation of FUS in cytoplasm	Dormann et al., 2010
	*fus*	Overexpression (R495X)	-	Inhibition of nuclear import, aggregation of FUS in cytoplasm and perinuclear stress granules	Bosco et al., 2010
	*fus*	Mutant (R521H)	Aberrant motor behavior	Decreased axonal length	Kabashi et al., 2011
	*fus*	Overexpression (R521H)	Aberrant motor behavior	Decreased axonal length	Kabashi et al., 2011
	*fus*	Morphant	Decreased swimming duration	Increase in motor neuron excitability, reduction in NMJ synaptic fidelity	Armstrong and Drapeau, 2013b
	*fus*	Mutant	Decreased swimming duration	Increase in motor neuron excitability, reduction in NMJ synaptic fidelity, early death	Bourefis et al., 2020
	*fus*	CRISPR-Cas9 mediated knockout	-	-	Lebedeva et al., 2017
*C9ORF72*	*c9orf72*	Morphant	Impairment of touch evoked escape response, decrease in swimming distance and velocity	Decrease in motor axon length and aberrant branching	Ciura et al., 2013; Yeh et al., 2018
	*c9orf72*	Stable loss of function mutant	Locomotion defects	Motor neuron loss, muscle atrophy, NMJ abnormalities, defects in synaptic vesicle trafficking and release, early death	Butti et al., 2021
	*c9orf72*	Stable transgenic (89x GGGGCC repeats)	Locomotion defects	Formation of RNA foci or dipeptide repeat (DPR), activation of heat shock response, motor neuron loss, muscle atrophy, high mortality	Shaw et al., 2018
	*c9orf72*	Overexpression (3x, 4x, 10x, 35x, 70x, and 90x GGGGCC repeats)	-	Motor axonopathy only from 35x repeat	Swinnen et al., 2018
	*c9orf72*	Overexpression (8x, 38x, and 72x GGGGCC repeats)	-	Increase in apoptotic cell death in 38x and 72x embryos	Lee et al., 2013
	*c9orf72*	Overexpression (2x or 80x GGGGCC repeats, with or without ATG codon)	-	Toxicity and pericardial edema in 80x embryos	Ohki et al., 2017
	*c9orf72*	Overexpression (1,000 GA or GR DPR)	Locomotion defects	-	Swaminathan et al., 2018
	*c9orf72*	Stable transgenic (100 GR DPR)	Reduced swimming	Morphological defects	Swaminathan et al., 2018

Antisense morpholino mediated knockdown of *tardbp* led to a reduction in axon length and abnormal branching followed by impairment of tail coiling ability and loss of touch evoked escape response (Kabashi et al., 2010). Similar effects were observed by the overexpression of three separate *TARDBP* mutations (A315T, A382T, and G348C) in the zebrafish embryos along with the loss of 50% of the motor neurons (Kabashi et al., 2010). Studies by Armstrong and Drapeau (2013a) and Lissouba et al. (2018) found that the G348C mutation in larvae impaired swimming and affected NMJ structures. As the single deletion of one ortholog of zebrafish compensated for the loss of one *TARDBP* ortholog with a splice variant of the other, Hewamadduma et al. (2013), Schmid et al. (2013), and Bose et al. (2019a) generated homozygous double mutants *(tardbp-/-; tardbpl-/-)* targeting both orthologs. These mutants had issues with spinal cord motor neuron growth, muscle degeneration, mispatterned vasculature, and early death. Interestingly, Bose et al. (2019a) using CRISPR-Cas9 mutants observed additional pathological features including morphological, locomotor, and synaptic dysfunction and structural abnormalities at the NMJ (Table 2). A recent study by Campanari et al. (2021) showed that motor defects and NMJ abnormalities in the zebrafish knockdown model of *tardbp* are associated with a reduction in the expression of acetylcholinesterase (AChE). AChE is vital for the functioning of NMJ. The overexpression of human AChE partially rescued the motor and NMJ deficits, potentially serving as the limiting factor regulating the muscle-motor neuron connection. Another potential mechanism of TDP-43 pathogenesis is related to abnormalities in neurofilament light (NEFL) expression and splicing. Reduction in the expression of NEFL in motor neurons is associated with ALS symptoms. Interestingly, TDP-43 binds with NEFL mRNA at its 3'UTR (MJ, 2007). A recent study in zebrafish revealed that MO-mediated knockdown of TDP-43 disrupts the splicing of NEFL zebrafish ortholog *neflb*, as the overexpression of one isoform of *neflb* rescued the reduction in the motor neuron axon length of TDP-43 knockdown embryos, while the other isoform exacerbated the phenotypes (Demy et al., 2020).

Zebrafish models have been instrumental in the study of *FUS*, a gene linked to ALS (Table 2). Dormann et al. (2010) and Bosco et al. (2010) recapitulated the prominent features of FUS pathology by injecting human mutant *FUS* mRNA P525L and R495X, respectively in the zebrafish embryos. The overexpression of the P525L mutation in the spinal cord neurons and muscle cells leads to inhibition of the nuclear import and aggregation of FUS in the cytoplasm. The R495X mutation exhibited similar phenotypes, with oxidative stress or heat shock treatments causing *fus* mutants to accumulate in perinuclear stress granules. Both knockdown of zebrafish *fus* and overexpression of the human *FUS* mutation R521H induced motor phenotype characterized by a reduced axonal length and aberrant motor behavior in embryos (Kabashi et al., 2011). Armstrong and Drapeau (2013b), observed related phenotypes in larvae including increased motor neuron excitability, reduced synaptic fidelity of the NMJ and shorter swimming duration. Homozygous mutants with a deletion mutation in *fus*, which leads to reduced production of Fus protein, also exhibited similar phenotypes, including reduced life span. These effects were linked to altered expression of acetylcholine receptor (AChR) subunits and histone deacetylase 4, paralleling denervation, and reinnervation processes seen in ALS (Bourefis et al., 2020). Contrastingly, a CRISPR knockout model of *fus* in zebrafish did not show any axonopathy and motor deficits, confirming that the effect of *fus* mutation is caused either due to its impaired loss of function or mislocalised gain of function (Lebedeva et al., 2017).

Researchers have been using zebrafish to study how a particular genetic change, known as the hexanucleotide repeat expansion in C9orf72, affects neuron development (Table 2). MO-mediated knockdown of *c9orf72* results in motor axonopathy and locomotion defects at larval stages as demonstrated by Ciura et al. (2013) and Yeh et al. (2018). Recently, a stable loss of function *c9orf72* zebrafish model has been developed. This model revealed that reduced C9orf72 expression causes motor neuron loss, locomotion defects, muscle atrophy, and abnormalities in the structure and function of NMJ, including impaired synaptic vesicle trafficking and release, culminating in early larval and adult mortality (Butti et al., 2021). Gain-of-function mutations in *c9orf72* lead to the formation of RNA foci or dipeptide repeat (DPR) proteins. Shaw et al. (2018) developed stable transgenic lines which expressed C9orf72 hexanucleotide repeat expansions. Interestingly, both mutants developed RNA foci and DPRs and exhibited features similar to those of loss-of-function mutants. These mutants also demonstrated that repeat expansions activate the heat shock response (HSR), leading to further progression of the disease phenotypes in the fish. Swinnen et al. (2018) revealed that while both, RNA toxicity and DPR toxicity induced motor axonopathy, repeat RNA toxicity is independent of DPR formation. Lee et al. (2013) showed that RNA foci also contribute to apoptotic cell death in zebrafish embryos and determined that RNA toxicity may affect RNA processing, contributing to neuron loss and degeneration. Investigating the neurotoxicity of the poly-Gly-Ala (poly-GA) DPR, most frequently found in ALS patient brains, Ohki et al. (2017) confirmed its detrimental effects using a transgenic approach. In a contrasting study, Swaminathan et al. (2018) found GR DPR to be the most toxic while GA to be the least toxic. Notably, their study revealed that the expression of 1,000 repeats of any DPR, whether GA or GR, led to locomotion defects in zebrafish. They then developed a transgenic line stably expressing 100 GR repeats and confirmed that GR DPRs are also neurotoxic and particularly affect motor neuron function.

In conclusion, extensive research utilizing zebrafish models has significantly advanced our understanding of ALS, revealing key insights into the genetic and molecular underpinnings of this devastating disease. Studies have elucidated the roles of critical genes like *SOD1, TARDBP, FUS*, and *C9ORF72* in ALS pathogenesis, highlighting mechanisms such as toxic gain-of-function, RNA toxicity, and the impact of genetic mutations on motor neuron health and functionality. The zebrafish models have been pivotal in demonstrating how these genetic alterations lead to motor neuron degeneration, muscular atrophy, and locomotion defects, closely mirroring the human condition. Future research directions should focus on disentangling the precise cellular pathways affected by these mutations, further exploring the potential of gene therapy, and identifying novel therapeutic targets. This could involve detailed studies on RNA processing and synaptic function in motor neurons, as well as the development of more advanced zebrafish models to simulate the disease's progression and response to potential treatments. The ultimate goal remains to translate these findings into effective clinical therapies for ALS, potentially halting or reversing the course of this currently incurable condition.

## 6 Spinal muscular atrophy

Spinal Muscular Atrophy (SMA) is an autosomal recessive neurodegenerative disorder. It is characterized by the loss of lower alpha motor neurons, progressive muscle weakness and premature death (D'Amico et al., 2011). The primary cause of SMA is mutations in the survival motor neuron 1 *(SMN1)* gene. This gene encodes a protein that is ubiquitously expressed in both the nucleus and the cytoplasm. The SMN1 protein has a crucial role in the assembly of small nuclear ribonucleoproteins (snRNPs), necessary for pre-mRNA splicing (Lefebvre et al., 1995; Burghes and Beattie, 2009; Koh et al., 2021). Interestingly, it also possess a potential role in the transport of mRNA in neurons (Burghes and Beattie, 2009). The severity of SMA progression hinges upon the amount of protein produced by survival motor neuron 2 *(SMN2)*, which varies among individuals due to differences in copy numbers. *SMN2* is nearly identical to *SMN1* and also encodes for the SMN protein. However, a nucleotide difference at position 6 in exon 7 alters the splicing pattern of the *SMN2*. This alteration results in the production of an unstable truncated protein (Pellizzoni et al., 1998; Lorson et al., 1999; Monani et al., 1999; Lorson and Androphy, 2000). Based on the amount of residual functional protein present, SMA is categorized into four different types, with a single copy in type I (severe) to four or more copies in type IV (adult onset) (Zerres et al., 1997; D'Amico et al., 2011).

To advance the understanding of SMA's pathophysiology, researchers developed various zebrafish models (Table 3). In these models, MO reduces Smn levels in zebrafish embryos. This reduction leads to defects in motor axon outgrowth and pathfinding (McWhorter et al., 2003; Winkler et al., 2005; Carrel et al., 2006; Oprea et al., 2008). Further research by Carrel et al. (2006) revealed that the *smn* transient morphants were rescued by wild type human SMN RNA. However, RNA from *smn2* and human *SMN* mutations did not rescue the phenotypes. They also discovered that *smn*'s role in motor neurons is independent of its function in small nuclear ribonucleoprotein (snRNP) assembly. In a contrasting study, Winkler et al. (2005) showed that silencing of *smn* expression in zebrafish arrested embryonic development and caused motor axon degeneration. Both phenotypes were rescued by the injection of purified snRNPs. This rescue suggests a direct link between snRNP assembly and motor axon degeneration in SMA patients. In a more recent study, Laird et al. (2016) used an miR-mediated knockdown of *smn1* to develop a transgenic zebrafish line. This line allowed for spatio-temporal control of *smn1* expression. Their research demonstrated that the knockdown of *smn1* in motor neurons alone was sufficient to reproduce the key characteristics of SMA.

**Table 3:** Characterization of the disease phenotypes in zebrafish model of SMA.

**Human genes**	**Zebrafish genes**	**Zebrafish modeling**	**Motor deficit**	**Other phenotype**	**References**
*SMN*	*smn*	Morphant	-	Defect in motor axon outgrowth and pathfinding	McWhorter et al., 2003; Winkler et al., 2005; Carrel et al., 2006; Oprea et al., 2008
	*smn*	Morphant	-	Hampering of embryonic development, motor axon degeneration	Winkler et al., 2005
	*smn*	miR-mediated knockdown	Loss of motor function	Scoliosis-like body deformities, weight loss, muscle atrophy, reduction in the number of motor neurons, abnormal motor neuron development, premature death	Laird et al., 2016
	*smn*	Mutant	-	Small size, early death within 2-3 weeks post fertilization, decrease in SV2 protein in neuromuscular system	Boon et al., 2009
	*smn*	Morphant	-	Defects in motor axon outgrowth and bioenergetic pathways	Boyd et al., 2017
	*smn*	Mutant	Locomotion defects	Shorter motor axons and dendrites with fewer branches, less filopodia, shorter average half-life	Hao et al., 2013
	*smn*	Mutant	Reduced locomotion	NMJ deficiencies, muscle atrophy, early death, normal motor neuron development	Tay et al., 2021

Despite the ubiquitous expression of *smn*, its mutation-induced specificity for motor neurons remains unclear. To investigate *smn*'s precise role in motor neurons, Boon et al. (2009) generated three *smn* mutations by TILING. Two of the mutations, *smnY262stop* and *smnL265stop* truncated exon 7, while the third one was a missense mutation complementary to a severe form of SMA in humans. Homozygous mutants with these mutant alleles were smaller in size and died within 2-3 weeks post fertilization. Notably, this was paralleled by a decrease in the synaptic vesicle protein SV2 in the neuromuscular system. Boon et al. (2009) created a *smn* mutant line which carries the human SMN protein exclusively in motoneurons. They found that the introduction of wild-type human SMN rescues the SV2 decrease in motor neurons. These findings established that *smn* is required to maintain the presynaptic integrity in the motor neurons. Spiró et al. (2016) used a different approach to study *smn*. They established a zebrafish culture system of GFP marked motorneurons (Spiró et al., 2016). They observed increased Smn levels in motorneurons and that additional Smn protein supply is necessary for proper axon formation. This highlights the dependence of motorneurons on Smn and may explain their increased vulnerability to *smn* mutations. Interestingly, not all motor neuron pools are equally affected in SMA. Mouse studies suggest that this selective vulnerability largely depends on the basal bioenergetic profile. Boyd et al. (2017), using zebrafish model, showed that defects in bioenergetic profiling are characteristic of SMA pathology (Boyd et al., 2017). They discovered that the targeting of genes involved in bioenergetic pathways rescues motor neuron outgrowth defects. This finding underscores the importance of such pathways as potential therapeutic targets.

Numerous studies established that *smn* mutations cause motor axon defects, yet it remains unclear why the motor units become dysfunctional. To address this issue, Hao et al. (2013) conducted a comprehensive analysis of motor neuron development in early embryonic stages in zebrafish system under low Smn levels. They observed that motor axons and dendrites were shorter and had fewer branches. The mutants possessed few filopodia, displayed locomotion defects, and had a shorter average half-life. This study confirms the role of Smn in the proper development of motor neuron axon and dendrites during early embryonic stages.

For a model to be accurately representative of SMA, it is crucial to replicate the dysfunction of *SMN1* gene and include presence of *SMN2* gene. However, *SMN2* is exclusive to humans (Rochette et al., 2001), making the creation of an SMA model challenging. The most effective approach involves introducing the human *SMN2 (hSMN2)* gene as a transgene in an animal with a mutated *smn1* gene. Hao et al. (2011) pioneered this method by generating a transgenic fish carrying the *hSMN2* gene. When this transgene was introduced into an *smn* mutant background, it rescued the neuromuscular presynaptic SV2 defect and imporved their survival rate. In a groundbreaking study, Tay et al. (2021) attempted to simulate the intermediate type SMA in zebrafish. Using homology directed repair (HDR), they developed a zebrafish model possessing intermediate levels of Smn protein (Tay et al., 2021). Initially, the Smn protein levels were normal, but as they decreased significantly over time, the mutants exhibited neuromuscular junction (NMJ) deficiencies, reduced locomotion, muscular atrophy, and early adult mortality. This occurred despite normal motor neuron development and maintenance in the early stages.

In conclusion, the comprehensive studies on SMA using zebrafish models have significantly advanced our understanding of the disease's pathophysiology. These models have elucidated the critical role of the *SMN1* and *SMN2* genes in motor neuron development and maintenance, highlighting the consequences of *SMN* mutations at the molecular and cellular levels. The research demonstrates a clear link between *SMN* mutations and motor axon defects, presynaptic integrity, and the necessity of SMN protein for motor neuron survival. Despite these advances, the exact mechanism by which *SMN* mutations selectively impact motor neurons remains elusive. Future research should focus on unraveling this specificity, perhaps by investigating the bioenergetic profiles of motor neurons and exploring the potential of bioenergetic pathways as therapeutic targets. Additionally, the development of more representative models, particularly those simulating intermediate and adult-onset SMA types, could provide further insights into the disease's progression and potential interventions.

## 7 Drug screening in zebrafish model of MNDs

**Figure 3:**
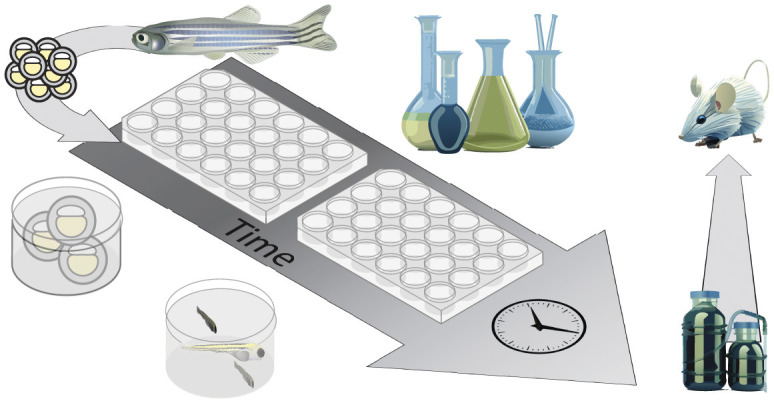
Diagrammatic representation of the strategy for high-throughput drug screening in zebrafish. Zebrafish embryos are subjected to large scale inexpensive chemical screen in 96-well plates and are assessed for changes in behavior, drug toxicity, metabolic profile, and other phenotypes. The lead compounds are either first tested in mammalian model or straight away undergo clinical trials. Only the compounds which are approved for safety and efficacy by the food and drug administration (FDA) are tested directly in human patients.

The escalating number of MND patient casualties annually hastens the demand for developing suitable drug targets. The aforementioned morphological, physiological, and behavioral attributes (see Section 2) make zebrafish ideal for large-scale *in vivo* bioassays in which embryos and larvae are exposed to a library of small molecules in multi-well plates (Figure 3). Furthermore, these assays enable assessments in the whole animal and the study of drug effects at the cellular or tissue level (Zon and Peterson, 2005; MacRae and Peterson, 2015; Patton et al., 2021). This is achievable through a combination of gene editing techniques with cell or tissue-specific reporters. Such studies pave the way for the development of novel drugs along with the remodeling of the available ones for personalized use. In the past few decades, zebrafish HSP mutants have been treated with several pharmacologically active molecules to discover the appropriate drug targets for effectively treating HSP patients (Table 4). Fassier et al. (2010) showed that treatment of *atl1* morphant embryos with dorsomorphin, a pharmacological inhibitor of the BMP receptor kinase activity, was sufficient to rescue the locomotion and spinal motor axon defects. Similarly, Song et al. (2013) demonstrated the effectiveness of dorsomorphin in attenuating the over-activation of BMP signaling in *pnpla6* morphants, suggesting its potential for clinical trials in treating spastic paraplegia (SPG) phenotypes caused by the disturbances in BMP signaling. Supporting this, Mao et al. (2015) also found dorsomorphin to be effective in rescuing the phenotypic defects in *solute carrier family 33 member 1 (slc33a1)* knockdown zebrafish, caused by the upregulated BMP receptor type 1A (BMPR1A) activity. In studies focusing on ER stress and proteotoxicity, compounds like phenazine, methylene blue, N-acetyl-cysteine, guanabenz, and salubrinal partially rescued the locomotion defects, microtubule defects, and oxidative stress in *spast* mutants (Julien et al., 2016). Oleic acid, an ER stress modulator, was found to alleviate motility defects and reduces ER stress in *seipin* N88S mutant larvae, which exhibit reduced triglyceride levels and impaired swimming activity (Hölttä-Vuori et al., 2013).

**Table 4:** List of the compounds tested in zebrafish models of HSP, ALS, and SMA with their effect on the disease phenotype.

**Disease**	**Target gene in zebrafish**	**Compounds**	**Effects on phenotype**	**References**
HSP	*atl1*	Dorsomorphin	Inhibition of BMP signaling, rescues locomotion and spinal motor axon defects	Fassier et al., 2010
	*pnpla6*	Dorsomorphin	Inhibition of BMP signaling	Song et al., 2013
	*slc33a1*	Dorsomorphin	Rescue motor axon phenotypes	Mao et al., 2015
	*spast*	Phenazine, methylene blue, N-acetyl-cysteine, guanabenz and salubrinal	Partially rescues locomotion and microtubule defects and oxidative stress	Julien et al., 2016
	*seipin*	Oleic acid	Rescues motility defects and reduces ER stress in larvae	Hölttä-Vuori et al., 2013
	*spatacsin*	Miglustat	Reduces paralysis and correct GM2 staining in larvae	Boutry et al., 2018
	*amfr*	Simvastatin (SMV) and atorvastatin (ATV)	Improved length, axon branching defects and escape response	Deng et al., 2023
	*mct8*	Thyroid hormone analogs and clemastine	Rescue neurological phenotypes and hypomyelination	Zada et al., 2014, 2016
	*atp13a2*	N-acetylcysteine and furaltadone	Rescue increased Mn2+ sensitivity	Heins-Marroquin et al., 2019
ALS	*sod1*	Riluzole and apomorphine	Reduces early neuronal stress response	McGown et al., 2013
	*sod1*	Riluzole	Reduces spinal neuron excitability, rescue behavioral phenotypes, improve deficits in motor nerve circuitry development	Benedetti et al., 2016
	*sod1*	Olesoxime	Protection against oxidative stress	Da Costa et al., 2014
	*sod1*	protein disulphide isomerase (PDI) and BMC ((+/-)-trans-1,2-Bis (2-mercaptoacetamido) cyclohexane)	Rescue impaired movement	Parakh et al., 2020
	*sod1*	iron complex of an amphiphilic corrole (1-Fe)	Increase locomotor activity	Soll et al., 2021
	*sod1*	Pimozide	Reduces motor deficits	Patten et al., 2017
	*sod1*	TRVA242	Rescue locomotor, motor neuron and NMJ synaptic deficits	Bose et al., 2019b
	*sod1*	Mdivi-1	Blocks Drp1 activity and prevent motor axon degeneration	Choi et al., 2020
	*sod1*	Combination of Ciprofloxacin and Celecoxib	Improve motor performance, recover motor neuron impairment, microglia morphology, and abnormalties in NMJ structure	Goldshtein et al., 2020
	*sod1*	Telbivudine	Rescue axonal and motor phenotypes in a dose dependent manner	DuVal et al., 2019
	*tardbp*	Methylene blue	Reduces oxidative stress and neuronal toxicity	Vaccaro et al., 2012
	*tardbp*	Salubrinal, guanabenz, phenazine, and methylene blue	Protect against oxidative stress and paralysis	Vaccaro et al., 2013
	*tardbp*	Terbutaline sulfate	Rescue axon and NMJ degeneration	Paik et al., 2015
	*tardbp*	FPL 64176 or Bay K 8644	Rescue motor phenotypes and NMJ abnormalities	Armstrong and Drapeau, 2013a
	*tardbp*	Pimozide	Reduces motor deficits and stabilizes neuromuscular transmission	Patten et al., 2017
	*tardbp*	TRVA242	Rescue locomotor, motor neuron and NMJ synaptic deficits	Bose et al., 2019b
	*tardbp*	Mdivi-1	Blocks Drp1 activity and prevent motor axon degeneration	Choi et al., 2020
	*tardbp*	Overexpression of phosphoglycerate kinase 1 (PGK1)	Increase in distance moved during touch evoked escape response (TEER)	Chaytow et al., 2022
	*tardbp*	Terazosin	Increase in distance moved during touch evoked escape response (TEER)	Chaytow et al., 2022
	*tardbp*	Combination of Ciprofloxacin and Celecoxib	Improve motor performance	Goldshtein et al., 2020
	*fus*	Methylene blue	Reduces oxidative stress and neuronal toxicity	Vaccaro et al., 2012
	*fus*	Pimozide	Reduces motor deficits	Patten et al., 2017
	*c9orf72*	Trolox	Reduces oxidative stress and poly-GR-medaited toxicity	Riemslagh et al., 2021
	*c9orf72*	TRVA242	Rescue locomotor, motor neuron and NMJ synaptic deficits	Bose et al., 2019b
	*c9orf72*	Overexpression of phosphoglycerate kinase 1 (PGK1)	Increase in distance moved during touch evoked escape response (TEER)	Chaytow et al., 2022
	*c9orf72*	Sodium phenylbutyrate and BET Bromodomain inhibitors	Reduce nucleolar stress	Corman et al., 2019
	*sqstm1/p62*	Rapamycin	Rescues locomotor phenotype	Lattante et al., 2015
SMA	*smn*	Tetrahydroindoles (SBL-154, SBL-185, SBL-190)	Rescue motor axon defects	Gassman et al., 2013
	*smn*	Quercetin	Inhibition of β-catenin, alleviate neuromuscular pathology	Wishart et al., 2014
	*smn*	Terazosin	Rescue motor axon defects	Boyd et al., 2017
	*smn*	Neurocalcin delta (NCALD)	Mitigate endocytosis and SMA pathological defects	Riessland et al., 2017
	*smn*	Apicidin, MG132, IOX1, Dipyridamole	Rescue motor axon length	Oprişoreanu et al., 2021
	*smn*	IOX1	Rescue aberrant presynaptic neuromuscular synapse	Oprişoreanu et al., 2021
	*smn*	Dipyridamole	Rescue aberrant presynaptic neuromuscular synapse morphology and axon growth defects	Oprişoreanu et al., 2021

Miglustat, known for reducing GM2 ganglioside levels in mouse models of Sandhoff disease (Jeyakumar et al., 1999), was shown by Boutry et al. (2018) to correct gangliosides accumulation in lysosomes and motor phenotypes in zebrafish with inhibited *spatacsin* expression. They further showed that inhibition of *spatacsin* expression in zebrafish larvae leads to a motor phenotype and strong GM2 staining in telencephalon. Interestingly, treatment with miglustat reduced paralysis and corrected GM2 staining in the larvae. This finding suggests potential novel therapeutics for MNDs linked to lysosomal dysfunction. Additionally, a limited number of drugs have also been tested for some rare forms of HSP using zebrafish as a model. Use of FDA-approved statins- simvastatin (SMV) and atorvastatin (ATV) improved length, axon branching defects and touch-evoked escape response in *amfra* homozygous mutant larvae (Deng et al., 2023). In their studies, Zada et al. (2014, 2016) demonstrated that thyroid hormone analogs and clemastine partially rescued neurological phenotypes and hypomyelination in *mct8-/-* mutants. Their results open up the prospects of exploring the potential of zebrafish for large scale drug screening for identifying more compounds of therapeutic potential. Another intriguing study demonstrated how the drugs tested in yeast can also rescue the phenotypes in zebrafish. Heins-Marroquin et al. (2019) developed a zebrafish model of *atp13a2* whose mutations are casually linked to HSP. They found that partial or complete loss of *atp13a2* increases Mn2+ sensitivity in zebrafish. The effects were reduced by two drugs, N-acetylcysteine and furaltadone, which were already tested in yeast for their therapeutic potential.

Zebrafish mutants for ALS disease genes have been extensively used to test the potential of several drugs for the treatment of this devastating MND (Table 4). McGown et al. (2013) showed that exposure to riluzole, the only approved drug for ALS and apomorphine, a nuclear factor erythroid 2-related factor 2 (NRF2) activator, reduced early neuronal stress response in the transgenic mutant *sod1* fish. Benedetti et al. (2016) supported these findings by demonstrating riluzole's effectiveness in reducing spinal neuron excitability, reverting behavioral phenotypes, and improving the deficits in motor nerve circuitry development in *sod1* mutant zebrafish by modulating the pacemaker sodium current (INaP). Additionally, the antioxidant olesoxime was found to protect the T70I *sod1* zebrafish embryos against oxidative stress (Da Costa et al., 2014). The oxidoreductase activity of protein disulphide isomerase (PDI) and BMC ((+/-)-trans-1,2-Bis (2-mercaptoacetamido) cyclohexane) also improves motor function in zebrafish *sod1* mutants (Parakh et al., 2020). The iron complex of an amphiphilic corrole (1-Fe) enhanced the locomotor activity in the *sod1* mutants (Soll et al., 2021).

Methylene blue has also effectively reduced oxidative stress and neuronal toxicity in zebrafish models of *tdp-43* and *fus* (Vaccaro et al., 2012). Vaccaro et al. (2013) tested salubrinal, guanabenz and phenazine, and methylene blue, finding that all four compounds reduced ER stress, protecting *tdp-43* mutants against oxidative stress and paralysis. Riemslagh et al. (2021) recently showed that use of Trolox reduces oxidative stress which in turn fully suppresses the poly-GR toxicity in zebrafish embryos with abnormal motor neuron morphology caused by the microinjection of poly-GR RNA. Terbutaline sulfate, a drug commonly used for asthma treatment, was effective in rescuing the TDP-43 pathological symptoms, including axon and NMJ degeneration (Paik et al., 2015). Findings from the study of Armstrong and Drapeau (2013a) revealed that hallmark features of ALS, including motor phenotypes and NMJ abnormalities, can also be rescued by the chronic treatment of *tdp-43* mutant zebrafish larvae with two L-type calcium channel agonists, FPL 64176, or Bay K 8644. Patten et al. (2017) established that pimozide, a neuroleptic compound, works as a T-type calcium channel antagonist, which reduces motor defects and stabilizes neuromuscular transmission in the *tdp-43* mutant zebrafish. It similarly benefits human *SOD1*-G93A and *FUS*-R521H Tg zebrafish and reduces motor deficits in them as well. A small molecule derivative of pimozide, TRVA242 has also shown effectiveness in improving the locomotor, motorneuron and NMJ synaptic deficits of *tdp-43, sod1*, and *c9orf72* mutant zebrafish models (Bose et al., 2019b). Mdivi-1 blocks Drp1 activity and effectively prevents motor axon degeneration in G93A *sod1* and Q331K *tdp-43* mutant zebrafish embryos (Choi et al., 2020). Recently, Chaytow et al. (2022) showed that in *tdp-43* mutant larvae, overexpression of phosphoglycerate kinase 1 (PGK1) or treatment with terazosin significantly increased the distance traveled during tail-touch evoked escape response (TEER). Interestingly, overexpression of PGK1 is also effective in treating *c9orf72* mutant larvae, but not terazosin (Chaytow et al., 2022).

In a recent study, Goldshtein et al. (2020) demonstrated that administration of a combination of Ciprofloxacin and Celecoxib improved the motor performance in *sod1* and *tdp-43* mutants. This treatment also rectified impaired motor neuron and microglia morphology and abnormalties in the NMJ structure of *sod1* mutants. Recently, Telbivudine, a uracil-like nucleoside compound, has been found to interact with the tryptophan residue at position 32 (W32) of Sod1. This residue is known for contributing to the Sod1-induced toxicity. Thereby, Telbivudine effectively rescues the axonal and motor phenotypes induced by the wild type or mutant *sod1* in a dose-dependent manner (DuVal et al., 2019). Corman et al. (2019), through chemical screening, identified sodium phenylbutyrate and bromodomain and extra-terminal domain (BET) inhibitors as promising drugs for alleviating nucleolar stress caused by dipeptide repeat proteins (DPRs) in developing zebrafish embryos. Moreover, mutations in some of the rare genes associated with ALS, i.e., sequestosome-1 *(SQSTM1)* cause behavioral and axonal anomalies and upregulation of mammalian target of rapamycin (mTOR) signaling in zebrafish embryos. Interestingly, exposure to rapamycin, a well-known inhibitor of the mTOR pathway, rescued the locomotor phenotype in the mutant fish (Lattante et al., 2015).

While a number of studies have been conducted in the past for screening drugs for the treatment of HSP and ALS, the capacity of zebrafish to identify the therapeutic compounds for SMA has yet to be explored much (Table 4). Three of the tetrahydroindoles, usually known for altering the metabolism of amyloid precursor protein (APP), rescued motor axon defects in a zebrafish model of SMA (Gassman et al., 2013). In their study, Wishart et al. (2014) found that perturbations in ubiquitin homeostasis, including reduction in the levels of ubiquitin-like modifier activating enzyme 1 (UBA1), was sufficient to cause neuromuscular pathology in the zebrafish model of SMA (Wishart et al., 2014). Dysregulation of ubiquitylation pathways also caused the accumulation of β-catenin. Notably, inhibition of β-catenin by quercetin helped mitigate the neuromuscular pathology, thereby highlighting the importance of ubiquitin homeostasis and β-catenin as potential therapeutic targets for SMA. Boyd et al. (2017) highlighted the significance of bioenergetic pathways, particularly the bioenergetic protein phosphoglycerate kinase 1 (Pgk1), as a therapeutic target for SMA. They showed that treating Pgk1 with terazosin, a Food and Drug Administration (FDA)-approved drug, which binds and activates Pgk1, rescues motor axon defects in a zebrafish SMA model. Taking a different approach, Riessland et al. (2017) identified a reduction of the neuronal calcium sensor neurocalcin delta (NCALD) as a protective SMA modifier. They found that NCALD works as a negative regulator of endocytosis and its knockdown ameliorates endocytosis and SMA associated pathological defects in zebrafish (Riessland et al., 2017). Synaptic degeneration is a common feature of many neurodegenerative disorders including SMA, yet there are few drugs available to counteract the synaptic destabilization defects. Oprişoreanu et al. (2021) characterized an automated screening paradigm in zebrafish to identify compounds that stabilize the neuromuscular synapses. Out of 982 compounds tested, four (Apicidin, MG132, IOX1, and dipyridamole) significantly rescued motor axon length. IOX1 and dipyridamole effectively corrected abnormal presynaptic neuromuscular synapse morphology. Moreover, dipyridamole also ameliorated axonal growth defects in zebrafish SMA mutants (Oprişoreanu et al., 2021).

## 8 Conclusion

In conclusion, the use of zebrafish models has been invaluable in Motor Neuron Disease (MND) research. These models replicate the key features of human MNDs, providing the benefits of genetic tractability and high-throughput drug screening. However, merely having models that replicate disease phenotypes is insufficient for identifying potential treatments. To create more effective treatments, the cellular and molecular basis of neuronal degeneration has to be revealed. The initial step in this direction is to develop stable zebrafish MND models. In particular, replicating patient mutations would be highly beneficial. Contrarily, most existing studies have relied on transient approaches to target the genes in zebrafish, like MO-based mutagenesis. These approaches are often prone to create off-target effects that mask the real nature of the mutation. The advent of CRISPR-Cas9-mediated mutagenesis now makes it possible to develop stable mutants with pinpoint precision. CRISPR-Cas9's extreme efficacy enables us to generate exact copies of patient mutations, which can now be analyzed in great detail in the zebrafish model.

The development of patient mutations is a prerequisite to the analysis of the aforementioned cellular and molecular basis of neuronal degeneration. The significant similarity between human and zebrafish genes, both at genetic and functional level, allows us to trace the repercussions of the mutation through the different biological contexts, from molecular to neuronal and, finally, behavioral. For instance, in most Hereditary Spastic Paraplegia (HSP) subtypes, abnormalities in various pathways, such as axon transport, lipid metabolism, ER morphogenesis, endosomal trafficking, autophagy etc, are believed to contribute to the disease's pathology. Distinguishing which of these pathways are directly responsible for the disease and which are secondary effects is essential for a comprehensive understanding of the disease mechanisms and for the development of targeted therapies.

Additionally, it is worth noting that the vast majority of studies conducted to understand MNDs in zebrafish use larvae, with minimal research on adult specimens. Considering that many MNDs show late-onset symptoms, crucial genes might be overlooked in larval screens. To accurately study these late-onset disease phenotypes, we must develop methods for adult screening, both biochemically and behaviorally. Effective and interpretable behavioral screening necessitates further fundamental research into the behavioral patterns of adult zebrafish. Therefore, future research directions should include the development of stable genetic mutants, the establishment of screening techniques for adult zebrafish, and a comprehensive understanding of adult zebrafish behavior.

## Author contributions

VG: Conceptualization, Writing – original draft, Writing – review & editing. BG: Conceptualization, Supervision, Writing – original draft, Writing – review & editing.

## References

[B1] Al-Chalabi A. Van Den Berg L. H. Veldink J. (2017). Gene discovery in amyotrophic lateral sclerosis: implications for clinical management. Nat. Rev. Neurol. 13, 96–104. 10.1038/nrneurol.2016.182 27982040

[B2] Amsterdam A. Burgess S. Golling G. Chen W. Sun Z. Townsend K.. (1999). A large-scale insertional mutagenesis screen in zebrafish. Genes Dev. 13, 2713–2724. 10.1101/gad.13.20.2713 10541557 PMC317115

[B3] Amsterdam A. Nissen R. M. Sun Z. Swindell E. C. Farrington S. Hopkins N. (2004). Identification of 315 genes essential for early zebrafish development. Proc. Nat. Acad. Sci. 101, 12792–12797. 10.1073/pnas.0403929101 15256591 PMC516474

[B4] Armstrong G. A. B, and Drapeau, P.. (2013a). Calcium channel agonists protect against neuromuscular dysfunction in a genetic model of TDP-43 mutation in ALS. J. Neurosci. 33, 1741–1752. 10.1523/JNEUROSCI.4003-12.2013 23345247 PMC6618732

[B5] Armstrong G. A. B. Drapeau P. (2013b). Loss and gain of FUS function impair neuromuscular synaptic transmission in a genetic model of ALS. Hum. Mol. Genet. 22, 4282–4292. 10.1093/hmg/ddt278 23771027

[B6] Armstrong G. A. B. Liao M. You Z. Lissouba A. Chen B. E. Drapeau P. (2016). Homology directed knockin of point mutations in the zebrafish tardbp and fus genes in als using the crispr/cas9 system. PLoS ONE 11:e0150188. 10.1371/journal.pone.0150188 26930076 PMC4773037

[B7] Asakawa K. Kawakami K. (2008). Targeted gene expression by the gal4-uas system in zebrafish. Dev. Growth Different. 50, 391–399. 10.1111/j.1440-169X.2008.01044.x 18482403

[B8] Auer T. O. Xiao T. Bercier V. Gebhardt C. Duroure K. Concordet J.-P.. (2015). Deletion of a kinesin i motor unmasks a mechanism of homeostatic branching control by neurotrophin-3. Elife 4:e05061. 10.7554/eLife.05061 26076409 PMC4467164

[B9] Babin P. J. Goizet C. Raldua D. (2014). Zebrafish models of human motor neuron diseases: advantages and limitations. Prog. Neurobiol. 118, 36–58. 10.1016/j.pneurobio.2014.03.001 24705136

[B10] Balciunas D. Wangensteen K. J. Wilber A. Bell J. Geurts A. Sivasubbu S.. (2006). Harnessing a high cargo-capacity transposon for genetic applications in vertebrates. PLoS Genet. 2:e169. 10.1371/journal.pgen.0020169 17096595 PMC1635535

[B11] Balendra R. Isaacs A. M. (2018). C9orf72-mediated als and ftd: multiple pathways to disease. Nat. Rev. Neurol. 14, 544–558. 10.1038/s41582-018-0047-2 30120348 PMC6417666

[B12] Basnet R. Zizioli D. Taweedet S. Finazzi D. Memo M. (2019). Zebrafish larvae as a behavioral model in neuropharmacology. Biomedicines 7:23. 10.3390/biomedicines7010023 30917585 PMC6465999

[B13] Benedetti L. Ghilardi A. Rottoli E. De Maglie M. Prosperi L. Perego C.. (2016). INAP selective inhibition reverts precocious inter-and motorneurons hyperexcitability in the sod1-g93r zebrafish ALS model. Sci. Rep. 6:24515. 10.1038/srep24515 27079797 PMC4832213

[B14] Berning B. A. Walker A. K. (2019). The pathobiology of tdp-43 c-terminal fragments in als and ftld. Front. Neurosci. 13:335. 10.3389/fnins.2019.00335 31031584 PMC6470282

[B15] Blackstone C. (2012). Cellular pathways of hereditary spastic paraplegia. Annu. Rev. Neurosci. 35, 25–47. 10.1146/annurev-neuro-062111-150400 22540978 PMC5584684

[B16] Blackstone C. (2018). Hereditary spastic paraplegia. Handb. Clin. Neurol. 148, 633–652. 10.1016/B978-0-444-64076-5.00041-7 29478605

[B17] Blader P. Strähle U. (2000). Zebrafish developmental genetics and central nervous system development. Hum. Mol. Genet. 9, 945–951. 10.1093/hmg/9.6.945 10767318

[B18] Boon K.-L. Xiao S. McWhorter M. L. Donn T. Wolf-Saxon E. Bohnsack M. T.. (2009). Zebrafish survival motor neuron mutants exhibit presynaptic neuromuscular junction defects. Hum. Mol. Genet. 18, 3615–3625. 10.1093/hmg/ddp310 19592581 PMC2742401

[B19] Bosco D. A. Lemay N. Ko H. K. Zhou H. Burke C. Kwiatkowski T. J. Jr. (2010). Mutant fus proteins that cause amyotrophic lateral sclerosis incorporate into stress granules. Hum. Mol. Genet. 19, 4160–4175. 10.1093/hmg/ddq335 20699327 PMC2981014

[B20] Bose P. Armstrong G. A. Drapeau P. (2019a). Neuromuscular junction abnormalities in a zebrafish loss-of-function model of tdp-43. J. Neurophysiol. 121, 285–297. 10.1152/jn.00265.2018 30461368

[B21] Bose P. Tremblay E. Maios C. Narasimhan V. Armstrong G. A. Liao M.. (2019b). The novel small molecule trva242 stabilizes neuromuscular junction defects in multiple animal models of amyotrophic lateral sclerosis. Neurotherapeutics 16, 1149–1166. 10.1007/s13311-019-00765-w 31342410 PMC6985319

[B22] Bourefis A.-R. Campanari M.-L. Buee-Scherrer V. Kabashi E. (2020). Functional characterization of a FUS mutant zebrafish line as a novel genetic model for ALS. Neurobiol. Dis. 142:104935. 10.1016/j.nbd.2020.104935 32380281

[B23] Boutry M. Branchu J. Lustremant C. Pujol C. Pernelle J. Matusiak R.. (2018). Inhibition of lysosome membrane recycling causes accumulation of gangliosides that contribute to neurodegeneration. Cell Rep. 23, 3813–3826. 10.1016/j.celrep.2018.05.098 29949766 PMC6045775

[B24] Boyd P. J. Tu W.-Y. Shorrock H. K. Groen E. J. Carter R. N. Powis R. A.. (2017). Bioenergetic status modulates motor neuron vulnerability and pathogenesis in a zebrafish model of spinal muscular atrophy. PLoS Genet. 13:e1006744. 10.1371/journal.pgen.1006744 28426667 PMC5417717

[B25] Brown R. H. Al-Chalabi A. (2017). Amyotrophic lateral sclerosis. New Engl. J. Med. 377, 162–172. 10.1056/NEJMra1603471 28700839

[B26] Bruni G. Lakhani P. Kokel D. (2014). Discovering novel neuroactive drugs through high-throughput behavior-based chemical screening in the zebrafish. Front. Pharmacol. 5:92695. 10.3389/fphar.2014.00153 25104936 PMC4109429

[B27] Brustein E. Saint-Amant L. Buss R. R. Chong M. McDearmid J. R. Drapeau P. (2003). Steps during the development of the zebrafish locomotor network. J. Physiol. Paris 97, 77–86. 10.1016/j.jphysparis.2003.10.009 14706693

[B28] Burgess H. A. Granato M. (2007). Modulation of locomotor activity in larval zebrafish during light adaptation. J. Exper. Biol. 210, 2526–2539. 10.1242/jeb.003939 17601957

[B29] Burghes A. H. Beattie C. E. (2009). Spinal muscular atrophy: why do low levels of survival motor neuron protein make motor neurons sick? Nat. Rev. Neurosci. 10, 597–609. 10.1038/nrn2670 19584893 PMC2853768

[B30] Buss R. R. Drapeau P. (2001). Synaptic drive to motoneurons during fictive swimming in the developing zebrafish. J. Neurophysiol. 86, 197–210. 10.1152/jn.2001.86.1.197 11431502

[B31] Butti Z. Pan Y. E. Giacomotto J. Patten S. A. (2021). Reduced c9orf72 function leads to defective synaptic vesicle release and neuromuscular dysfunction in zebrafish. Commun. Biol. 4:792. 10.1038/s42003-021-02302-y 34172817 PMC8233344

[B32] Campanari M.-L. Marian A. Ciura S. Kabashi E. (2021). Tdp-43 regulation of ache expression can mediate als-like phenotype in zebrafish. Cells 10:221. 10.3390/cells10020221 33499374 PMC7911940

[B33] Campbell P. D. Shen K. Sapio M. R. Glenn T. D. Talbot W. S. Marlow F. L. (2014). Unique function of kinesin kif5a in localization of mitochondria in axons. J. Neurosci. 34, 14717–14732. 10.1523/JNEUROSCI.2770-14.2014 25355224 PMC4212069

[B34] Carrel T. L. McWhorter M. L. Workman E. Zhang H. Wolstencroft E. C. Lorson C.. (2006). Survival motor neuron function in motor axons is independent of functions required for small nuclear ribonucleoprotein biogenesis. J. Neurosci. 26, 11014–11022. 10.1523/JNEUROSCI.1637-06.2006 17065443 PMC6674655

[B35] Chakrabarti S. Streisinger G. Singer F. Walker C. (1983). Frequency of γ-ray induced specific locus and recessive lethal mutations in mature germ cells of the zebrafish, brachydanio rerio. Genetics 103, 109–123. 10.1093/genetics/103.1.109 17246098 PMC1202016

[B36] Chang J. Lee S. Blackstone C.. (2014). Spastic paraplegia proteins spastizin and spatacsin mediate autophagic lysosome reformation. J. Clin. Invest. 124, 5249–5262. 10.1172/JCI77598 25365221 PMC4348974

[B37] Chaytow H. Carroll E. Gordon D. Huang Y.-T. Van Der Hoorn D. Smith H. L.. (2022). Targeting phosphoglycerate kinase 1 with terazosin improves motor neuron phenotypes in multiple models of amyotrophic lateral sclerosis. EBioMedicine 83:104202. 10.1016/j.ebiom.2022.104202 35963713 PMC9482929

[B38] Chia K. Klingseisen A. Sieger D. Priller J. (2022). Zebrafish as a model organism for neurodegenerative disease. Front. Mol. Neurosci. 15:940484. 10.3389/fnmol.2022.940484 36311026 PMC9606821

[B39] Choi S. Y. Lee J.-H. Chung A.-Y. Jo Y. Shin J.-H. Park H.-C.. (2020). Prevention of mitochondrial impairment by inhibition of protein phosphatase 1 activity in amyotrophic lateral sclerosis. Cell Death Dis. 11:888. 10.1038/s41419-020-03102-8 33087694 PMC7578657

[B40] Choi T.-Y. Choi T.-I. Lee Y.-R. Choe S.-K. Kim C.-H. (2021). Zebrafish as an animal model for biomedical research. Exper. Molec. Med. 53, 310–317. 10.1038/s12276-021-00571-5 33649498 PMC8080808

[B41] Ciura S. Lattante S. Le Ber I. Latouche M. Tostivint H. Brice A.. (2013). Loss of function of c9orf72 causes motor deficits in a zebrafish model of amyotrophic lateral sclerosis. Ann. Neurol. 74, 180–187. 10.1002/ana.23946 23720273

[B42] Clemen C. S. Tangavelou K. Strucksberg K.-H. Just S. Gaertner L. Regus-Leidig H.. (2010). Strumpellin is a novel valosin-containing protein binding partner linking hereditary spastic paraplegia to protein aggregation diseases. Brain 133, 2920–2941. 10.1093/brain/awq222 20833645

[B43] Clift D. Richendrfer H. Thorn R. J. Colwill R. M. Creton R. (2014). High-throughput analysis of behavior in zebrafish larvae: effects of feeding. Zebrafish 11, 455–461. 10.1089/zeb.2014.0989 25153037 PMC4172468

[B44] Corman A. Jung B. Häggblad M. Bräutigam L. Lafarga V. Lidemalm L.. (2019). A chemical screen identifies compounds limiting the toxicity of c9orf72 dipeptide repeats. Cell Chem. Biol. 26, 235–243. 10.1016/j.chembiol.2018.10.020 30527999

[B45] Da Costa M. M. Allen C. E. Higginbottom A. Ramesh T. Shaw P. J. McDermott C. J. (2014). A new zebrafish model produced by tilling of sod1-related amyotrophic lateral sclerosis replicates key features of the disease and represents a tool for in vivo therapeutic screening. Dis. Models Mechan. 7, 73–81. 10.1242/dmm.012013 24092880 PMC3882050

[B46] D'Amico A. Mercuri E. Tiziano F. D. Bertini E. (2011). Spinal muscular atrophy. Orphanet J. Rare Dis. 6, 1–10. 10.1186/1750-1172-6-71 22047105 PMC3231874

[B47] DAmore A. Tessa A. Naef V. Bassi M. T. Citterio A. Romaniello R.. (2020). Loss of ap4s1 in zebrafish leads to neurodevelopmental defects resembling spastic paraplegia 52. Ann. Clin. Transl. Neurol. 7, 584–589. 10.1002/acn3.51018 32216065 PMC7187712

[B48] Davis Jr G. Farel P. B. (1990). Mauthner cells maintain their lumbar projection in adult frog. Neurosci. Lett. 113, 139–143. 10.1016/0304-3940(90)90293-I 2377313

[B49] Demy D. L. Campanari M. L. Munoz-Ruiz R. Durham H. D. Gentil B. J. Kabashi E. (2020). Functional characterization of neurofilament light splicing and misbalance in zebrafish. Cells 9, 1238. 10.3390/cells9051238 32429483 PMC7291018

[B50] Deng R. Medico-Salsench E. Nikoncuk A. Ramakrishnan R. Lanko K. Kühn N. A.. (2023). *AMFR* dysfunction causes autosomal recessive spastic paraplegia in human that is amenable to statin treatment in a preclinical model. Acta Neuropathol. 146, 353–368. 10.1007/s00401-023-02579-9 37119330 PMC10328903

[B51] Desai N. Olney R. (2009). “Neuromuscular diseases,” in Clinical Adult Neurology (New York: Demos Medical Publishing), 307–333.

[B52] Dion P. A. Daoud H. Rouleau G. A. (2009). Genetics of motor neuron disorders: new insights into pathogenic mechanisms. Nat. Rev. Genet. 10, 769–782. 10.1038/nrg2680 19823194

[B53] Dormann D. Rodde R. Edbauer D. Bentmann E. Fischer I. Hruscha A.. (2010). Als-associated fused in sarcoma (fus) mutations disrupt transportin-mediated nuclear import. EMBO J. 29, 2841–2857. 10.1038/emboj.2010.143 20606625 PMC2924641

[B54] Dosch R. Wagner D. S. Mintzer K. A. Runke G. Wiemelt A. P. Mullins M. C. (2004). Maternal control of vertebrate development before the midblastula transition: mutants from the zebrafish I. Dev. Cell 6, 771–780. 10.1016/j.devcel.2004.05.002 15177026

[B55] Drapeau P. Saint-Amant L. Buss R. R. Chong M. McDearmid J. R. Brustein E. (2002). Development of the locomotor network in zebrafish. Prog. Neurobiol. 68, 85–111. 10.1016/S0301-0082(02)00075-8 12450489

[B56] Driever W. Solnica-Krezel L. Schier A. Neuhauss S. Malicki J. Stemple D.. (1996). A genetic screen for mutations affecting embryogenesis in zebrafish. Development 123, 37–46. 10.1242/dev.123.1.37 9007227

[B57] DuVal M. G. Hinge V. K. Snyder N. Kanyo R. Bratvold J. Pokrishevsky E.. (2019). Tryptophan 32 mediates sod1 toxicity in a in vivo motor neuron model of ALS and is a promising target for small molecule therapeutics. Neurobiol. Dis. 124, 297–310. 10.1016/j.nbd.2018.11.025 30528257

[B58] Eaton R. C. Bombardieri R. A. Meyer D. L. (1977). The mauthner-initiated startle response in teleost fish. J. Exper. Biol. 66, 65–81. 10.1242/jeb.66.1.65 870603

[B59] Ekker S. C. (2008). Zinc finger-based knockout punches for zebrafish genes. Zebrafish 5, 121–123. 10.1089/zeb.2008.9988 18554175 PMC2849655

[B60] Elsayed L. E. Eltazi I. Z. Ahmed A. E. Stevanin G. (2021). Insights into clinical, genetic, and pathological aspects of hereditary spastic paraplegias: a comprehensive overview. Front. Molec. Biosci. 8:690899. 10.3389/fmolb.2021.690899 34901147 PMC8662366

[B61] Errico A. Ballabio A. Rugarli E. I. (2002). Spastin, the protein mutated in autosomal dominant hereditary spastic paraplegia, is involved in microtubule dynamics. Hum. Mol. Genet. 11, 153–163. 10.1093/hmg/11.2.153 11809724

[B62] Evans K. Keller C. Pavur K. Glasgow K. Conn B. Lauring B. (2006). Interaction of two hereditary spastic paraplegia gene products, spastin and atlastin, suggests a common pathway for axonal maintenance. Proc. Nat. Acad. Sci. 103, 10666–10671. 10.1073/pnas.0510863103 16815977 PMC1502289

[B63] Faber D. Korn H. (1978). Electrophysiology of the mauthner cell: basic properties, synaptic mechanisms, and associated networks. Neurobiol. Mauthner Cell 10, 47–131.

[B64] Fard M. A. F. Rebelo A. P. Buglo E. Nemati H. Dastsooz H. Gehweiler I.. (2019). Truncating mutations in ubap1 cause hereditary spastic paraplegia. Am. J. Human Genet. 104, 767–773. 10.1016/j.ajhg.2019.03.001 30929741 PMC6451742

[B65] Fassier C. Hutt J. A. Scholpp S. Lumsden A. Giros B. Nothias F.. (2010). Zebrafish atlastin controls motility and spinal motor axon architecture via inhibition of the bmp pathway. Nat. Neurosci. 13, 1380–1387. 10.1038/nn.2662 20935645

[B66] Fetcho J. R. (1992). The spinal motor system in early vertebrates and some of its evolutionary changes. Brain Behav. Evol. 40, 82–97. 10.1159/000113905 1422809

[B67] Fink J. K. (2006). Hereditary spastic paraplegia. Curr. Neurol. Neurosci. Rep. 6, 65–76. 10.1007/s11910-996-0011-1 16469273

[B68] Fink J. K. (2014). “Hereditary spastic paraplegia: clinical principles and genetic advances,” in Seminars in neurology (Thieme Medical Publishers), 293–305. 10.1055/s-0034-1386767 25192507

[B69] Finsterer J. Löscher W. Quasthoff S. Wanschitz J. Auer-Grumbach M. Stevanin G. (2012). Hereditary spastic paraplegias with autosomal dominant, recessive, x-linked, or maternal trait of inheritance. J. Neurol. Sci. 318, 1–18. 10.1016/j.jns.2012.03.025 22554690

[B70] Foster L. A. Salajegheh M. K. (2019). Motor neuron disease: pathophysiology, diagnosis, and management. Am. J. Med. 132, 32–37. 10.1016/j.amjmed.2018.07.012 30075105

[B71] Friesema E. C. Ganguly S. Abdalla A. Fox J. E. M. Halestrap A. P. Visser T. J. (2003). Identification of monocarboxylate transporter 8 as a specific thyroid hormone transporter. J. Biol. Chem. 278, 40128–40135. 10.1074/jbc.M300909200 12871948

[B72] Fulwiler C. Gilbert W. (1991). Zebrafish embryology and neural development. Curr. Opin. Cell Biol. 3, 988–991. 10.1016/0955-0674(91)90118-I 1814370

[B73] Galatolo D. Tessa A. Filla A. Santorelli F. M. (2018). Clinical application of next generation sequencing in hereditary spinocerebellar ataxia: increasing the diagnostic yield and broadening the ataxia-spasticity spectrum. a retrospective analysis. Neurogenetics 19, 1–8. 10.1007/s10048-017-0532-6 29209898

[B74] Gan-Or Z. Bouslam N. Birouk N. Lissouba A. Chambers D. B. Vérièpe J.. (2016). Mutations in capn1 cause autosomal-recessive hereditary spastic paraplegia. Am. J. Hum. Genet. 98, 1038–1046. 10.1016/j.ajhg.2016.04.002 27153400 PMC4863665

[B75] Garg V. André S. Giraldo D. Heyer L. Göpfert M. C. Dosch R.. (2022). A markerless pose estimator applicable to limbless animals. Front. Behav. Neurosci. 16:819146. 10.3389/fnbeh.2022.819146 35418841 PMC8997243

[B76] Gassman A. Hao L. T. Bhoite L. Bradford C. L. Chien C.-B. Beattie C. E.. (2013). Small molecule suppressors of drosophila kinesin deficiency rescue motor axon development in a zebrafish model of spinal muscular atrophy. PLoS ONE 8:e74325. 10.1371/journal.pone.0074325 24023935 PMC3762770

[B77] Gerlai R. (2010). High-throughput behavioral screens: the first step towards finding genes involved in vertebrate brain function using zebrafish. Molecules 15, 2609–2622. 10.3390/molecules15042609 20428068 PMC6257226

[B78] Goldshtein H. Muhire A. Petel Legare V. Pushett A. Rotkopf R. Shefner J. M.. (2020). Efficacy of ciprofloxacin/celecoxib combination in zebrafish models of amyotrophic lateral sclerosis. Ann. Clin. Transl. Neurol. 7, 1883–1897. 10.1002/acn3.51174 32915525 PMC7545590

[B79] Gore S. V. Kakodkar R. Del Rosario Hernández T. Edmister S. T. Creton R. (2023). Zebrafish larvae position tracker (z-lap tracker): a high-throughput deep-learning behavioral approach for the identification of calcineurin pathway-modulating drugs using zebrafish larvae. Sci. Rep. 13:3174. 10.1038/s41598-023-30303-w 36823315 PMC9950053

[B80] Grad L. I. Rouleau G. A. Ravits J. Cashman N. R. (2017). Clinical spectrum of amyotrophic lateral sclerosis (als). Cold Spring Harb. Perspect. Med. 7:a024117. 10.1101/cshperspect.a024117 28003278 PMC5538408

[B81] Gray J. (1933). Studies in animal locomotion: I. The movement of fish with special reference to the EEL. J. Exper. Biol. 10, 88–104. 10.1242/jeb.10.1.88

[B82] Green J. Collins C. Kyzar E. J. Pham M. Roth A. Gaikwad S.. (2012). Automated high-throughput neurophenotyping of zebrafish social behavior. J. Neurosci. Methods 210, 266–271. 10.1016/j.jneumeth.2012.07.017 22884772

[B83] Gros-Louis F. Kriz J. Kabashi E. McDearmid J. Millecamps S. Urushitani M.. (2008). Als2 mrna splicing variants detected in ko mice rescue severe motor dysfunction phenotype in als2 knock-down zebrafish. Hum. Mol. Genet. 17, 2691–2702. 10.1093/hmg/ddn171 18558633

[B84] Haffter P. Granato M. Brand M. Mullins M. C. Hammerschmidt M. Kane D. A.. (1996). The identification of genes with unique and essential functions in the development of the zebrafish, danio rerio. Development 123, 1–36. 10.1242/dev.123.1.1 9007226

[B85] Halpern M. E. Rhee J. Goll M. G. Akitake C. M. Parsons M. Leach S. D. (2008). Gal4/uas transgenic tools and their application to zebrafish. Zebrafish 5, 97–110. 10.1089/zeb.2008.0530 18554173 PMC6469517

[B86] Hao L. T. Burghes A. H. Beattie C. E. (2011). Generation and characterization of a genetic zebrafish model of SMA carrying the human SMN2 gene. Mol. Neurodegener. 6, 1–9. 10.1186/1750-1326-6-24 21443782 PMC3080329

[B87] Hao L. T. Duy P. Q. Jontes J. D. Wolman M. Granato M. Beattie C. E. (2013). Temporal requirement for SMN in motoneuron development. Hum. Mol. Genet. 22, 2612–2625. 10.1093/hmg/ddt110 23459934 PMC3674802

[B88] Harding A. (1993). “Hereditary spastic paraplegias,” in Seminars in Neurology (Thieme Medical Publishers, Inc.), 333–336. 10.1055/s-2008-1041143 8146482

[B89] Harding A. E. (1984). The hereditary ataxias and related disorders. Clin. Neurol. Neurosurg. Monogr. 6, 57–70.

[B90] Hasani H. Sun J. Zhu S. I. Rong Q. Willomitzer F. Amor R.. (2023). Whole-brain imaging of freely-moving zebrafish. Front. Neurosci. 17:1127574. 10.3389/fnins.2023.1127574 37139528 PMC10150962

[B91] Hazan J. Fonknechten N. Mavel D. Paternotte C. Samson D. Artiguenave F.. (1999). Spastin, a new AAA protein, is altered in the most frequent form of autosomal dominant spastic paraplegia. Nat. Genet. 23, 296–303. 10.1038/15472 10610178

[B92] Heins-Marroquin U. Jung P. P. Cordero-Maldonado M. L. Crawford A. D. Linster C. L. (2019). Phenotypic assays in yeast and zebrafish reveal drugs that rescue *ATP13A2* deficiency. Brain Commun. 1:fcz019. 10.1093/braincomms/fcz019 32954262 PMC7425419

[B93] Hewamadduma C. A. Grierson A. J. Ma T. P. Pan L. Moens C. B. Ingham P. W.. (2013). Tardbpl splicing rescues motor neuron and axonal development in a mutant TARDBP zebrafish. Hum. Mol. Genet. 22, 2376–2386. 10.1093/hmg/ddt082 23427147 PMC3658164

[B94] Hölttä-Vuori M. Salo V. T. Ohsaki Y. Suster M. L. Ikonen E. (2013). Alleviation of seipinopathy-related ER stress by triglyceride storage. Hum. Mol. Genet. 22, 1157–1166. 10.1093/hmg/dds523 23250914

[B95] Howe K. Clark M. D. Torroja C. F. Torrance J. Berthelot C. Muffato M.. (2013). The zebrafish reference genome sequence and its relationship to the human genome. Nature 496, 498–503. 10.1038/nature12111 23594743 PMC3703927

[B96] Huang P. Xiao A. Tong X. Lin S. Zhang B. (2016). “Targeted mutagenesis in zebrafish by talens,” in TALENs: Methods and Protocols, 191–206. 10.1007/978-1-4939-2932-0_15 26443223

[B97] Hufnagel R. B. Arno G. Hein N. D. Hersheson J. Prasad M. Anderson Y.. (2015). Neuropathy target esterase impairments cause oliver-mcfarlane and laurence-moon syndromes. J. Med. Genet. 52, 85–94. 10.1136/jmedgenet-2014-102856 25480986 PMC8108008

[B98] Hussain M. (2023). YOLO-v1 to YOLO-v8, the rise of YOLO and its complementary nature toward digital manufacturing and industrial defect detection. Machines 11:677. 10.3390/machines11070677

[B99] Hwang W. Y. Fu Y. Reyon D. Maeder M. L. Tsai S. Q. Sander J. D.. (2013). Efficient genome editing in zebrafish using a CRISPR-CAS system. Nat. Biotechnol. 31, 227–229. 10.1038/nbt.2501 23360964 PMC3686313

[B100] Ingebretson J. J. Masino M. A. (2013). Quantification of locomotor activity in larval zebrafish: considerations for the design of high-throughput behavioral studies. Front. Neural Circ. 7:109. 10.3389/fncir.2013.00109 23772207 PMC3677137

[B101] Irion U. Krauss J. Nüsslein-Volhard C. (2014). Precise and efficient genome editing in zebrafish using the crispr/cas9 system. Development 141, 4827–4830. 10.1242/dev.115584 25411213 PMC4299274

[B102] Jardin N. Giudicelli F. Ten Martín D. Vitrac A. De Gois S. Allison R.. (2018). Bmp-and neuropilin 1-mediated motor axon navigation relies on spastin alternative translation. Development 145:dev162701. 10.1242/dev.162701 30082270 PMC6141775

[B103] Jeyakumar M. Butters T. D. Cortina-Borja M. Hunnam V. Proia R. L. Perry V. H.. (1999). Delayed symptom onset and increased life expectancy in sandhoff disease mice treated with *N*-butyldeoxynojirimycin. Proc. Nat. Acad. Sci. 96, 6388–6393. 10.1073/pnas.96.11.6388 10339597 PMC26891

[B104] Julien C. Lissouba A. Madabattula S. Fardghassemi Y. Rosenfelt C. Androschuk A.. (2016). Conserved pharmacological rescue of hereditary spastic paraplegia-related phenotypes across model organisms. Hum. Mol. Genet. 25, 1088–1099. 10.1093/hmg/ddv632 26744324 PMC4764191

[B105] Kabashi E. Bercier V. Lissouba A. Liao M. Brustein E. Rouleau G. A.. (2011). Fus and tardbp but not sod1 interact in genetic models of amyotrophic lateral sclerosis. PLoS Genet. 7:e1002214. 10.1371/journal.pgen.1002214 21829392 PMC3150442

[B106] Kabashi E. Lin L. Tradewell M. L. Dion P. A. Bercier V. Bourgouin P.. (2010). Gain and loss of function of als-related mutations of tardbp (tdp-43) cause motor deficits in vivo. Hum. Mol. Genet. 19, 671–683. 10.1093/hmg/ddp534 19959528

[B107] Kanagaraj P. Gautier-Stein A. Riedel D. Schomburg C. Cerdà J. Vollack N.. (2014). Souffle/spastizin controls secretory vesicle maturation during zebrafish oogenesis. PLoS Genet. 10:e1004449. 10.1371/journal.pgen.1004449 24967841 PMC4072560

[B108] Kaslin J. Panula P. (2001). Comparative anatomy of the histaminergic and other aminergic systems in zebrafish (danio rerio). J. Compar. Neurol. 440, 342–377. 10.1002/cne.1390 11745628

[B109] Kawakami K. (2004). “Transgenesis and gene trap methods in zebrafish by using the tol2 transposable element,” in Methods in Cell Biology (Elsevier), 201–222. 10.1016/S0091-679X(04)77011-9 15602913

[B110] Khaniani M. S. Derakhshan S. M. Abasalizadeh S. (2013). Prenatal diagnosis of spinal muscular atrophy: clinical experience and molecular genetics of SMN gene analysis in 36 cases. J. Prenat. Med. 7:32. 24175014 PMC3808942

[B111] Kiefel H. Bondong S. Hazin J. Ridinger J. Schirmer U. Riedle S.. (2012). L1cam: a major driver for tumor cell invasion and motility. Cell Adhes. Migr. 6, 374–384. 10.4161/cam.20832 22796939 PMC3478260

[B112] Kiernan M. C. Vucic S. Cheah B. C. Turner M. R. Eisen A. Hardiman O.. (2011). Amyotrophic lateral sclerosis. Lancet 377, 942–955. 10.1016/S0140-6736(10)61156-7 21296405

[B113] Kimmel C. B. Ballard W. W. Kimmel S. R. Ullmann B. Schilling T. F. (1995). Stages of embryonic development of the zebrafish. Dev. Dyn. 203, 253–310. 10.1002/aja.1002030302 8589427

[B114] Klebe S. Stevanin G. Depienne C. (2015). Clinical and genetic heterogeneity in hereditary spastic paraplegias: from spg1 to spg72 and still counting. Rev. Neurol. 171, 505–530. 10.1016/j.neurol.2015.02.017 26008818

[B115] Koh A. Sarusie M. V. Ohmer J. Fischer U. Winkler C. Wohland T. (2021). Fluorescence correlation spectroscopy reveals survival motor neuron oligomerization but no active transport in motor axons of a zebrafish model for spinal muscular atrophy. Front. Cell Dev. Biol. 9:639904. 10.3389/fcell.2021.639904 34458251 PMC8385639

[B116] Laale H. W. (1977). The biology and use of zebrafish, brachydanio rerio in fisheries research. A literature review. J. Fish Biol. 10, 121–173. 10.1111/j.1095-8649.1977.tb04049.x

[B117] Laird A. S. Mackovski N. Rinkwitz S. Becker T. S. Giacomotto J. (2016). Tissue-specific models of spinal muscular atrophy confirm a critical role of SMN in motor neurons from embryonic to adult stages. Hum. Mol. Genet. 25, 1728–1738. 10.1093/hmg/ddw044 26908606

[B118] Lattante S. de Calbiac H. Le Ber I. Brice A. Ciura S. Kabashi E. (2015). Sqstm1 knock-down causes a locomotor phenotype ameliorated by rapamycin in a zebrafish model of als/ftld. Hum. Mol. Genet. 24, 1682–1690. 10.1093/hmg/ddu580 25410659

[B119] Lattante S. Rouleau G. A. Kabashi E. (2013). Tardbp and fus mutations associated with amyotrophic lateral sclerosis: summary and update. Hum. Mutat. 34, 812–826. 10.1002/humu.22319 23559573

[B120] Lauder G. V. Tytell E. D. (2005). Hydrodynamics of undulatory propulsion. Fish Physiol. 23, 425–468. 10.1016/S1546-5098(05)23011-X

[B121] Lebedeva S. de Jesus Domingues A. M. Butter F. Ketting R. F. (2017). Characterization of genetic loss-of-function of fus in zebrafish. RNA Biol. 14, 29–35. 10.1080/15476286.2016.1256532 27898262 PMC5270537

[B122] Lee Y.-B. Chen H.-J. Peres J. N. Gomez-Deza J. Attig J. Štalekar M.. (2013). Hexanucleotide repeats in als/ftd form length-dependent rna foci, sequester rna binding proteins, and are neurotoxic. Cell Rep. 5, 1178–1186. 10.1016/j.celrep.2013.10.049 24290757 PMC3898469

[B123] Lefebvre S. Bürglen L. Reboullet S. Clermont O. Burlet P. Viollet L.. (1995). Identification and characterization of a spinal muscular atrophy-determining gene. Cell 80, 155–165. 10.1016/0092-8674(95)90460-3 7813012

[B124] Lemmens R. Van Hoecke A. Hersmus N. Geelen V. D'Hollander I. Thijs V.. (2007). Overexpression of mutant superoxide dismutase 1 causes a motor axonopathy in the zebrafish. Hum. Mol. Genet. 16, 2359–2365. 10.1093/hmg/ddm193 17636250

[B125] Li M. Zhao L. Page-McCaw P. S. Chen W. (2016). Zebrafish genome engineering using the crispr-cas9 system. Trends Genet. 32, 815–827. 10.1016/j.tig.2016.10.005 27836208 PMC5127170

[B126] Li Y. Zhang C. Peng G. (2023). Ap4s1 truncation leads to axonal defects in a zebrafish model of spastic paraplegia 52. Int. J. Dev. Neurosci. 83, 753–764. 10.1002/jdn.10303 37767851

[B127] Lieschke G. J. Currie P. D. (2007). Animal models of human disease: zebrafish swim into view. Nat. Rev. Genet. 8, 353–367. 10.1038/nrg2091 17440532

[B128] Lin P. Li J. Liu Q. Mao F. Li J. Qiu R.. (2008). A missense mutation in slc33a1, which encodes the acetyl-coa transporter, causes autosomal-dominant spastic paraplegia (spg42). Am. J. Hum. Genet. 83, 752–759. 10.1016/j.ajhg.2008.11.003 19061983 PMC2668077

[B129] Lin X. Jiang J.-Y. Hong D.-J. Lin K.-J. Li J.-J. Chen Y.-J.. (2023). Biallelic COQ4 variants in hereditary spastic paraplegia: Clinical and molecular characterization. Movem. Disor. 39, 152–163. 10.1002/mds.29664 38014483

[B130] Lin X. Su H.-Z. Dong E.-L. Lin X.-H. Zhao M. Yang C.. (2019). Stop-gain mutations in ubap1 cause pure autosomal-dominant spastic paraplegia. Brain 142, 2238–2252. 10.1093/brain/awz158 31203368

[B131] Linneberg C. Toft C. L. F. Kjaer-Sorensen K. Laursen L. S. (2019). L1cam-mediated developmental processes of the nervous system are differentially regulated by proteolytic processing. Sci. Rep. 9:3716. 10.1038/s41598-019-39884-x 30842511 PMC6403279

[B132] Lissouba A. Liao M. Kabashi E. Drapeau P. (2018). Transcriptomic analysis of zebrafish tdp-43 transgenic lines. Front. Mol. Neurosci. 11:463. 10.3389/fnmol.2018.00463 30618614 PMC6301209

[B133] Liu K. Petree C. Requena T. Varshney P. Varshney G. K. (2019a). Expanding the crispr toolbox in zebrafish for studying development and disease. Front. Cell Dev. Biol. 7:13. 10.3389/fcell.2019.00013 30886848 PMC6409501

[B134] Liu X. Guo X. Niu L. Li X. Sun F. Hu J.. (2019b). Atlastin-1 regulates morphology and function of endoplasmic reticulum in dendrites. Nat. Commun. 10:568. 10.1038/s41467-019-08478-6 30718476 PMC6362286

[B135] Loeffler J.-P. Picchiarelli G. Dupuis L. Gonzalez De Aguilar J.-L. (2016). The role of skeletal muscle in amyotrophic lateral sclerosis. Brain Pathol. 26, 227–236. 10.1111/bpa.12350 26780251 PMC8029271

[B136] Lorson C. L. Androphy E. J. (2000). An exonic enhancer is required for inclusion of an essential exon in the sma-determining gene SMN. Hum. Mol. Genet. 9, 259–265. 10.1093/hmg/9.2.259 10607836

[B137] Lorson C. L. Hahnen E. Androphy E. J. Wirth B. (1999). A single nucleotide in the SMN gene regulates splicing and is responsible for spinal muscular atrophy. Proc. Nat. Acad. Sci. 96, 6307–6311. 10.1073/pnas.96.11.6307 10339583 PMC26877

[B138] Lu B. Hwang M. Yong C. Moretti L. (2007). Zebrafish as a model system to screen radiation modifiers. Curr. Genomics 8, 360–369. 10.2174/138920207783406497 19412436 PMC2671721

[B139] MacRae C. A. Peterson R. T. (2015). Zebrafish as tools for drug discovery. Nature reviews Drug discovery 14, 721–731. 10.1038/nrd4627 26361349

[B140] Mao F. Li Z. Zhao B. Lin P. Liu P. Zhai M.. (2015). Identification and functional analysis of a slc 33 a 1: c. 339 t> g (p. s er113 a rg) variant in the original spg 42 family. Hum. Mutat. 36, 240–249. 10.1002/humu.22732 25402622

[B141] Martin E. Schüle R. Smets K. Rastetter A. Boukhris A. Loureiro J. L.. (2013). Loss of function of glucocerebrosidase gba2 is responsible for motor neuron defects in hereditary spastic paraplegia. Am. J. Hum. Genet. 92, 238–244. 10.1016/j.ajhg.2012.11.021 23332916 PMC3567271

[B142] Martin E. Yanicostas C. Rastetter A. Naini S. M. A. Maouedj A. Kabashi E.. (2012). Spatacsin and spastizin act in the same pathway required for proper spinal motor neuron axon outgrowth in zebrafish. Neurobiol. Dis. 48, 299–308. 10.1016/j.nbd.2012.07.003 22801083

[B143] Mathis A. Mamidanna P. Cury K. M. Abe T. Murthy V. N. Mathis M. W.. (2018). DeepLabCut: markerless pose estimation of user-defined body parts with deep learning. Nat. Neurosci. 21, 1281–1289. 10.1038/s41593-018-0209-y 30127430

[B144] Matsubara T. Oda M. Takahashi T. Watanabe C. Tachiyama Y. Morino H.. (2019). Amyotrophic lateral sclerosis of long clinical course clinically presenting with progressive muscular atrophy. Neuropathology 39, 47–53. 10.1111/neup.12523 30511354

[B145] McGown A. McDearmid J. R. Panagiotaki N. Tong H. Al Mashhadi S. Redhead N.. (2013). Early interneuron dysfunction in als: insights from a mutant sod1 zebrafish model. Ann. Neurol. 73, 246–258. 10.1002/ana.23780 23281025 PMC3608830

[B146] McWhorter M. L. Monani U. R. Burghes A. H. Beattie C. E. (2003). Knockdown of the survival motor neuron (SMN) protein in zebrafish causes defects in motor axon outgrowth and pathfinding. J. Cell Biol. 162, 919–932. 10.1083/jcb.200303168 12952942 PMC1761110

[B147] Melo U. S. Macedo-Souza L. I. Figueiredo T. Muotri A. R. Gleeson J. G. Coux G.. (2015). Overexpression of klc2 due to a homozygous deletion in the non-coding region causes spoan syndrome. Hum. Mol. Genet. 24, 6877–6885. 10.1093/hmg/ddv388 26385635 PMC6296331

[B148] Mero S. Salviati L. Leuzzi V. Rubegni A. Calderan C. Nardecchia F.. (2021). New pathogenic variants in coq4 cause ataxia and neurodevelopmental disorder without detectable coq 10 deficiency in muscle or skin fibroblasts. J. Neurol. 268, 3381–3389. 10.1007/s00415-021-10509-6 33704555

[B149] Miall R. C. (2022). “Cortical motor control,” in Neuroscience in the 21st Century: From Basic to Clinical (Springer), 1601–1622. 10.1007/978-3-030-88832-9_128

[B150] Mirat O. Sternberg J. R. Severi K. E. Wyart C. (2013). Zebrazoom: an automated program for high-throughput behavioral analysis and categorization. Front. Neural Circ. 7:107. 10.3389/fncir.2013.00107 23781175 PMC3679480

[B151] Miscevic F. Rotstein O. Wen X.-Y. (2012). Advances in zebrafish high content and high throughput technologies. Combinat. Chem. High Throughput Screen. 15, 515–521. 10.2174/138620712801619140 22497524

[B152] MJ S. (2007). Tdp43 is a human low molecular weight neurofilament (HNFL) mrna-binding protein. Mol. Cell. Neurosci. 35, 320–327. 10.1016/j.mcn.2007.03.007 17481916

[B153] Moens C. B. Donn T. M. Wolf-Saxon E. R. Ma T. P. (2008). Reverse genetics in zebrafish by tilling. Briefings Funct. Gen. Proteom. 7, 454–459. 10.1093/bfgp/eln046 19028802 PMC2899843

[B154] Monani U. R. Lorson C. L. Parsons D. W. Prior T. W. Androphy E. J. Burghes A. H.. (1999). A single nucleotide difference that alters splicing patterns distinguishes the SMA gene SMN1 from the copy gene SMN2. Hum. Mol. Genet. 8, 1177–1183. 10.1093/hmg/8.7.1177 10369862

[B155] Müller U. Stamhuis E. Videler J. (2000). Hydrodynamics of unsteady fish swimming and the effects of body size: comparing the flow fields of fish larvae and adults. J. Exper. Biol. 203, 193–206. 10.1242/jeb.203.2.193 10607529

[B156] Müller U. K. Van Leeuwen J. L. (2006). Undulatory fish swimming: from muscles to flow. Fish Fisher. 7, 84–103. 10.1111/j.1467-2979.2006.00210.x

[B157] Myers P. Z. (1985). Spinal motoneurons of the larval zebrafish. J. Compar. Neurol. 236, 555–561. 10.1002/cne.902360411 4056102

[B158] Myers P. Z. Eisen J. S. Westerfield M. (1986). Development and axonal outgrowth of identified motoneurons in the zebrafish. J. Neurosci. 6, 2278–2289. 10.1523/JNEUROSCI.06-08-02278.1986 3746410 PMC6568750

[B159] Naef V. Mero S. Fichi G. D'Amore A. Ogi A. Gemignani F.. (2019). Swimming in deep water: zebrafish modeling of complicated forms of hereditary spastic paraplegia and spastic ataxia. Front. Neurosci. 13:1311. 10.3389/fnins.2019.01311 31920481 PMC6914767

[B160] Ohki Y. Wenninger-Weinzierl A. Hruscha A. Asakawa K. Kawakami K. Haass C.. (2017). Glycine-alanine dipeptide repeat protein contributes to toxicity in a zebrafish model of c9orf72 associated neurodegeneration. Mol. Neurodegener. 12, 1–11. 10.1186/s13024-016-0146-8 28088213 PMC5237533

[B161] Oprea G. E. Krober S. McWhorter M. L. Rossoll W. Muller S. Krawczak M.. (2008). Plastin 3 is a protective modifier of autosomal recessive spinal muscular atrophy. Science 320, 524–527. 10.1126/science.1155085 18440926 PMC4908855

[B162] Oprişoreanu A. M. Smith H. L. Krix S. Chaytow H. Carragher N. O. Gillingwater T. H.. (2021). Automated in vivo drug screen in zebrafish identifies synapse-stabilising drugs with relevance to spinal muscular atrophy. Dis. Models Mech. 14:dmm047761. 10.1242/dmm.047761 33973627 PMC8106959

[B163] Orger M. B. de Polavieja G. G. (2017). Zebrafish behavior: opportunities and challenges. Annu. Rev. Neurosci. 40, 125–147. 10.1146/annurev-neuro-071714-033857 28375767

[B164] Paik H. Chung A.-Y. Park H.-C. Park R. W. Suk K. Kim J.. (2015). Repurpose terbutaline sulfate for amyotrophic lateral sclerosis using electronic medical records. Sci. Rep. 5:8580. 10.1038/srep08580 25739475 PMC4894399

[B165] Panula P. Sallinen V. Sundvik M. Kolehmainen J. Torkko V. Tiittula A.. (2006). Modulatory neurotransmitter systems and behavior: towards zebrafish models of neurodegenerative diseases. Zebrafish 3, 235–247. 10.1089/zeb.2006.3.235 18248264

[B166] Parakh S. Shadfar S. Perri E. R. Ragagnin A. M. Piattoni C. V. Fogolín M. B.. (2020). The redox activity of protein disulfide isomerase inhibits als phenotypes in cellular and zebrafish models. Iscience 23, 1–44. 10.1016/j.isci.2020.101097 32446203 PMC7240177

[B167] Parodi L. Coarelli G. Stevanin G. Brice A. Durr A. (2018). Hereditary ataxias and paraparesias: clinical and genetic update. Curr. Opin. Neurol. 31, 462–471. 10.1097/WCO.0000000000000585 29847346

[B168] Patten S. A. Aggad D. Martinez J. Tremblay E. Petrillo J. Armstrong G. A.. (2017). Neuroleptics as therapeutic compounds stabilizing neuromuscular transmission in amyotrophic lateral sclerosis. JCI insight 2:e97152. 10.1172/jci.insight.97152 29202456 PMC5752378

[B169] Patten S. A. Armstrong G. A. Lissouba A. Kabashi E. Parker J. A. Drapeau P. (2014). Fishing for causes and cures of motor neuron disorders. Dis. Models Mechan. 7, 799–809. 10.1242/dmm.015719 24973750 PMC4073270

[B170] Patton E. E. Zon L. I. Langenau D. M. (2021). Zebrafish disease models in drug discovery: from preclinical modelling to clinical trials. Nat. Rev. Drug Discov. 20, 611–628. 10.1038/s41573-021-00210-8 34117457 PMC9210578

[B171] Peggion C. Scalcon V. Massimino M. L. Nies K. Lopreiato R. Rigobello M. P.. (2022). Sod1 in als: taking stock in pathogenic mechanisms and the role of glial and muscle cells. Antioxidants 11:614. 10.3390/antiox11040614 35453299 PMC9032988

[B172] Pelkowski S. D. Kapoor M. Richendrfer H. A. Wang X. Colwill R. M. Creton R. (2011). A novel high-throughput imaging system for automated analyses of avoidance behavior in zebrafish larvae. Behav. Brain Res. 223, 135–144. 10.1016/j.bbr.2011.04.033 21549762 PMC3111907

[B173] Pellizzoni L. Kataoka N. Charroux B. Dreyfuss G. (1998). A novel function for SMN, the spinal muscular atrophy disease gene product, in pre-MRNA splicing. Cell 95, 615–624. 10.1016/S0092-8674(00)81632-3 9845364

[B174] Pérez-Escudero A. Vicente-Page J. Hinz R. C. Arganda S. De Polavieja G. G. (2014). idtracker: tracking individuals in a group by automatic identification of unmarked animals. Nat. Methods 11, 743–748. 10.1038/nmeth.2994 24880877

[B175] Ramesh T. Lyon A. N. Pineda R. H. Wang C. Janssen P. M. Canan B. D.. (2010). A genetic model of amyotrophic lateral sclerosis in zebrafish displays phenotypic hallmarks of motoneuron disease. Dis. Models Mechan. 3, 652–662. 10.1242/dmm.005538 20504969 PMC2931540

[B176] Randlett O. (2023). pi_tailtrack: a compact, inexpensive and open-source behaviour-tracking system for head-restrained zebrafish. J. Exper. Biol. 226:jeb246335. 10.1242/jeb.246335 37818550

[B177] Richendrfer H. Créton R. (2013). Automated high-throughput behavioral analyses in zebrafish larvae. JoVE 77:e50622. 10.3791/50622-v PMC3731428 23851916

[B178] Riemslagh F. W. Verhagen R. F. van der Toorn E. C. Smits D. J. Quint W. H. van der Linde H. C.. (2021). Reduction of oxidative stress suppresses poly-gr-mediated toxicity in zebrafish embryos. Dis. Models Mechan. 14:dmm049092. 10.1242/dmm.049092 34693978 PMC8649169

[B179] Riessland M. Kaczmarek A. Schneider S. Swoboda K. J. Löhr H. Bradler C.. (2017). Neurocalcin delta suppression protects against spinal muscular atrophy in humans and across species by restoring impaired endocytosis. Am. J. Hum. Genet. 100, 297–315. 10.1016/j.ajhg.2017.01.005 28132687 PMC5294679

[B180] Robberecht W. Philips T. (2013). The changing scene of amyotrophic lateral sclerosis. Nat. Rev. Neurosci. 14, 248–264. 10.1038/nrn3430 23463272

[B181] Robinson K. J. Yuan K. C. Don E. K. Hogan A. L. Winnick C. G. Tym M. C.. (2019). Motor neuron abnormalities correlate with impaired movement in zebrafish that express mutant superoxide dismutase 1. Zebrafish 16, 8–14. 10.1089/zeb.2018.1588 30300572 PMC6357263

[B182] Rochette C. Gilbert N. Simard L. (2001). SMN gene duplication and the emergence of the SMN2 gene occurred in distinct hominids: SMN2 is unique to homo sapiens. Hum. Genet. 108, 255–266. 10.1007/s004390100473 11354640

[B183] Roggenbuck J. Quick A. Kolb S. J. (2017). Genetic testing and genetic counseling for amyotrophic lateral sclerosis: an update for clinicians. Genet. Med. 19, 267–274. 10.1038/gim.2016.107 27537704

[B184] Romero-Ferrero F. Bergomi M. G. Hinz R. C. Heras F. J. de Polavieja G. G. (2019). Idtracker. AI: tracking all individuals in small or large collectives of unmarked animals. Nat. Methods 16, 179–182. 10.1038/s41592-018-0295-5 30643215

[B185] Russell K. L. Downie J. M. Gibson S. B. Tsetsou S. Keefe M. D. Duran J. A.. (2021). Pathogenic effect of tp73 gene variants in people with amyotrophic lateral sclerosis. Neurology 97, e225–e235. 10.1212/WNL.0000000000012285 34135078 PMC8302149

[B186] Saint-Amant L. Drapeau P. (1998). Time course of the development of motor behaviors in the zebrafish embryo. J. Neurobiol. 37, 622–632. 10.1002/(SICI)1097-4695(199812)37:4<622::AID-NEU10>3.0.CO;2-S 9858263

[B187] Sakowski S. A. Lunn J. S. Busta A. S. Oh S. S. Zamora-Berridi G. Palmer M.. (2012). Neuromuscular effects of g93a-sod1 expression in zebrafish. Mol. Neurodegener. 7, 1–15. 10.1186/1750-1326-7-44 22938571 PMC3506515

[B188] Sanderson C. M. Connell J. W. Edwards T. L. Bright N. A. Duley S. Thompson A.. (2006). Spastin and atlastin, two proteins mutated in autosomal-dominant hereditary spastic paraplegia, are binding partners. Hum. Mol. Genet. 15, 307–318. 10.1093/hmg/ddi447 16339213 PMC2443951

[B189] Schmid B. Hruscha A. Hogl S. Banzhaf-Strathmann J. Strecker K. van der Zee J.. (2013). Loss of als-associated tdp-43 in zebrafish causes muscle degeneration, vascular dysfunction, and reduced motor neuron axon outgrowth. Proc. Nat. Acad. Sci. 110, 4986–4991. 10.1073/pnas.1218311110 23457265 PMC3612625

[B190] Schmidt R. Strähle U. Scholpp S. (2013). Neurogenesis in zebrafish-from embryo to adult. Neural Dev. 8, 1–13. 10.1186/1749-8104-8-3 23433260 PMC3598338

[B191] Shaw M. P. Higginbottom A. McGown A. Castelli L. M. James E. Hautbergue G. M.. (2018). Stable transgenic c9orf72 zebrafish model key aspects of the ALS/FTD phenotype and reveal novel pathological features. Acta Neuropathol. Commun. 6, 1–16. 10.1186/s40478-018-0629-7 30454072 PMC6240957

[B192] Smits A. J. (2019). Undulatory and oscillatory swimming. J. Fluid Mech. 874:P1. 10.1017/jfm.2019.284

[B193] Soll M. Goldshtein H. Rotkopf R. Russek-Blum N. Gross Z. (2021). A synthetic sod/catalase mimic compound for the treatment of ALS. Antioxidants 10:827. 10.3390/antiox10060827 34067277 PMC8224677

[B194] Song Y. Wang M. Mao F. Shao M. Zhao B. Song Z.. (2013). Knockdown of PNPLA6 protein results in motor neuron defects in zebrafish. Dis. Models Mechan. 6, 404–413. 10.1242/dmm.009688 22996643 PMC3597022

[B195] Southgate L. Dafou D. Hoyle J. Li N. Kinning E. Critchley P.. (2010). Novel SPG11 mutations in Asian Kindreds and disruption of Spatacsin function in the zebrafish. Neurogenetics 11, 379–389. 10.1007/s10048-010-0243-8 20390432 PMC2944959

[B196] Spiró Z. Koh A. Tay S. See K. Winkler C. (2016). Transcriptional enhancement of SMN levels in motoneurons is crucial for proper axon morphology in zebrafish. Sci. Rep. 6:27470. 10.1038/srep27470 27273160 PMC4895340

[B197] Stepto A. Gallo J.-M. Shaw C. E. Hirth F. (2014). Modelling c9orf72 hexanucleotide repeat expansion in amyotrophic lateral sclerosis and frontotemporal dementia. Acta Neuropathol. 127, 377–389. 10.1007/s00401-013-1235-1 24366528

[B198] Streisinger G. Walker C. Dower N. Knauber D. Singer F. (1981). Production of clones of homozygous diploid zebra fish (brachydanio rerio). Nature 291, 293–296. 10.1038/291293a0 7248006

[B199] Suster M. L. Kikuta H. Urasaki A. Asakawa K. Kawakami K. (2009). “Transgenesis in zebrafish with the tol2 transposon system,” in Transgenesis Techniques: Principles and Protocols, 41–63. 10.1007/978-1-60327-019-9_3 19504063

[B200] Swaminathan A. Bouffard M. Liao M. Ryan S. Callister J. B. Pickering-Brown S. M.. (2018). Expression of c9orf72-related dipeptides impairs motor function in a vertebrate model. Hum. Mol. Genet. 27, 1754–1762. 10.1093/hmg/ddy083 29528390 PMC5932562

[B201] Swierczek N. A. Giles A. C. Rankin C. H. Kerr R. A. (2011). High-throughput behavioral analysis in C. ELEGANS. Nat. Methods 8, 592–598. 10.1038/nmeth.1625 21642964 PMC3128206

[B202] Swinnen B. Bento-Abreu A. Gendron T. F. Boeynaems S. Bogaert E. Nuyts R.. (2018). A zebrafish model for c9orf72 als reveals rna toxicity as a pathogenic mechanism. Acta Neuropathol. 135, 427–443. 10.1007/s00401-017-1796-5 29302778

[B203] Tay S. H. Ellieyana E. N. Le Y. Sarusie M. V. Grimm C. Ohmer J.. (2021). A novel zebrafish model for intermediate type spinal muscular atrophy demonstrates importance of SMN for maintenance of mature motor neurons. Hum. Mol. Genet. 30, 2488–2502. 10.1093/hmg/ddab212 34302176

[B204] Tesson C. Koht J. Stevanin G. (2015). Delving into the complexity of hereditary spastic paraplegias: how unexpected phenotypes and inheritance modes are revolutionizing their nosology. Hum. Genet. 134, 511–538. 10.1007/s00439-015-1536-7 25758904 PMC4424374

[B205] Tiryaki E. Horak H. A. (2014). Als and other motor neuron diseases. Lifelong Learn. Neurol. 20, 1185–1207. 10.1212/01.CON.0000455886.14298.a4 25299277

[B206] Udvadia A. J. Linney E. (2003). Windows into development: historic, current, and future perspectives on transgenic zebrafish. Dev. Biol. 256, 1–17. 10.1016/S0012-1606(02)00083-0 12654288

[B207] Uribe-Salazar J. M. Kaya G. Sekar A. Weyenberg K. Ingamells C. Dennis M. Y. (2022). Evaluation of crispr gene-editing tools in zebrafish. BMC Genom. 23, 1–16. 10.1186/s12864-021-08238-1 34986794 PMC8734261

[B208] Vaccaro A. Patten S. A. Aggad D. Julien C. Maios C. Kabashi E.. (2013). Pharmacological reduction of ER stress protects against tdp-43 neuronal toxicity in vivo. Neurobiol. Dis. 55, 64–75. 10.1016/j.nbd.2013.03.015 23567652

[B209] Vaccaro A. Patten S. A. Ciura S. Maios C. Therrien M. Drapeau P.. (2012). Methylene blue protects against TDP-43 and FUS neuronal toxicity in C. elegans and D. rerio. PLoS ONE 7:e42117. 10.1371/journal.pone.0042117 22848727 PMC3407135

[B210] Valdmanis P. N. Meijer I. A. Reynolds A. Lei A. MacLeod P. Schlesinger D.. (2007). Mutations in the kiaa0196 gene at the spg8 locus cause hereditary spastic paraplegia. Am. J. Hum. Genet. 80, 152–161. 10.1086/510782 17160902 PMC1785307

[B211] Vaz F. M. McDermott J. H. Alders M. Wortmann S. B. Kölker S. Pras-Raves M. L.. (2019). Mutations in pcyt2 disrupt etherlipid biosynthesis and cause a complex hereditary spastic paraplegia. Brain 142, 3382–3397. 10.1093/brain/awz291 31637422 PMC6821184

[B212] Voisard P. Diofano F. Glazier A. A. Rottbauer W. Just S. (2022). Crispr/cas9-mediated constitutive loss of vcp (valosin-containing protein) impairs proteostasis and leads to defective striated muscle structure and function in vivo. Int. J. Mol. Sci. 23:6722. 10.3390/ijms23126722 35743185 PMC9223409

[B213] Wagner M. Osborn D. P. Gehweiler I. Nagel M. Ulmer U. Bakhtiari S.. (2019). Bi-allelic variants in rnf170 are associated with hereditary spastic paraplegia. Nat. Commun. 10:4790. 10.1038/s41467-019-12620-9 31636353 PMC6803694

[B214] Walker C. Streisinger G. (1983). Induction of mutations by γ-rays in pregonial germ cells of zebrafish embryos. Genetics 103, 125–136. 10.1093/genetics/103.1.125 17246099 PMC1202017

[B215] Walter T. Couzin I. D. (2021). Trex, a fast multi-animal tracking system with markerless identification, and 2D estimation of posture and visual fields. Elife 10:e64000. 10.7554/eLife.64000 33634789 PMC8096434

[B216] Wang W.-C. Brehm P. (2017). A gradient in synaptic strength and plasticity among motoneurons provides a peripheral mechanism for locomotor control. Curr. Biol. 27, 415–422. 10.1016/j.cub.2016.12.010 28111148 PMC5310826

[B217] Wardle C. Videler J. Altringham J. (1995). Tuning in to fish swimming waves: body form, swimming mode and muscle function. J. Exper. Biol. 198, 1629–1636. 10.1242/jeb.198.8.1629 9319534

[B218] Wasel O. Freeman J. L. (2020). Chemical and genetic zebrafish models to define mechanisms of and treatments for dopaminergic neurodegeneration. Int. J. Mol. Sci. 21:5981. 10.3390/ijms21175981 32825242 PMC7503535

[B219] Wen H. Eckenstein K. Weihrauch V. Stigloher C. Brehm P. (2020). Primary and secondary motoneurons use different calcium channel types to control escape and swimming behaviors in zebrafish. Proc. Nat. Acad. Sci. 117, 26429–26437. 10.1073/pnas.2015866117 33020266 PMC7585033

[B220] Westerfield M. McMurray J. V. Eisen J. S. (1986). Identified motoneurons and their innervation of axial muscles in the zebrafish. J. Neurosci. 6, 2267–2277. 10.1523/JNEUROSCI.06-08-02267.1986 3746409 PMC6568761

[B221] Wienholds E. Schulte-Merker S. Walderich B. Plasterk R. H. (2002). Target-selected inactivation of the zebrafish RAG1 gene. Science. 297, 99–102. 10.1126/science.1071762 12098699

[B222] Wiessner M. Maroofian R. Ni M.-Y. Pedroni A. Müller J. S. Stucka R.. (2021). Biallelic variants in hpdl cause pure and complicated hereditary spastic paraplegia. Brain 144, 1422–1434. 10.1093/brain/awab041 33970200 PMC8219359

[B223] Will U. (1986). Mauthner neurons survive metamorphosis in anurans: a comparative HRP study on the cytoarchitecture of mauthner neurons in amphibians. J. Compar. Neurol. 244, 111–120. 10.1002/cne.902440109 3081602

[B224] Winkler C. Eggert C. Gradl D. Meister G. Giegerich M. Wedlich D.. (2005). Reduced u SNRNP assembly causes motor axon degeneration in an animal model for spinal muscular atrophy. Genes Dev. 19, 2320–2330. 10.1101/gad.342005 16204184 PMC1240041

[B225] Wishart T. M. Mutsaers C. A. Riessland M. Reimer M. M. Hunter G. Hannam M. L.. (2014). Dysregulation of ubiquitin homeostasis and β-catenin signaling promote spinal muscular atrophy. J. Clin. Invest. 124, 1821–1834. 10.1172/JCI71318 24590288 PMC3973095

[B226] Wood J. D. Landers J. A. Bingley M. McDermott C. J. Thomas-McArthur V. Gleadall L. J.. (2006). The microtubule-severing protein spastin is essential for axon outgrowth in the zebrafish embryo. Hum. Mol. Genet. 15, 2763–2771. 10.1093/hmg/ddl212 16893913

[B227] Yeh T.-H. Liu H.-F. Li Y.-W. Lu C.-S. Shih H.-Y. Chiu C.-C.. (2018). C9orf72 is essential for neurodevelopment and motility mediated by cyclin g1. Exp. Neurol. 304, 114–124. 10.1016/j.expneurol.2018.03.002 29522758

[B228] Zada D. Tovin A. Lerer-Goldshtein T. Appelbaum L. (2016). Pharmacological treatment and bbb-targeted genetic therapy for mct8-dependent hypomyelination in zebrafish. Dis. Models Mechan. 9, 1339–1348. 10.1242/dmm.027227 27664134 PMC5117236

[B229] Zada D. Tovin A. Lerer-Goldshtein T. Vatine G. D. Appelbaum L. (2014). Altered behavioral performance and live imaging of circuit-specific neural deficiencies in a zebrafish model for psychomotor retardation. PLoS Genet. 10:e1004615. 10.1371/journal.pgen.1004615 25255244 PMC4177677

[B230] Zerres K. Rudnik-Schöneborn S. Forrest E. Lusakowska A. Borkowska J. Hausmanowa-Petrusewicz I. (1997). A collaborative study on the natural history of childhood and juvenile onset proximal spinal muscular atrophy (type ii and iii sma): 569 patients. J. Neurol. Sci. 146, 67–72. 10.1016/S0022-510X(96)00284-5 9077498

[B231] Zivony-Elboum Y. Westbroek W. Kfir N. Savitzki D. Shoval Y. Bloom A.. (2012). A founder mutation in vps37a causes autosomal recessive complex hereditary spastic paraparesis. J. Med. Genet. 49, 462–472. 10.1136/jmedgenet-2012-100742 22717650

[B232] Zon L. I. Peterson R. T. (2005). In vivo drug discovery in the zebrafish. Nat. Rev. Drug Disc. 4, 35–44. 10.1038/nrd1606 15688071

